# Once upon a Time, There Was a Piece of Wood: Present Knowledge and Future Perspectives in Fungal Deterioration of Wooden Cultural Heritage in Terrestrial Ecosystems and Diagnostic Tools

**DOI:** 10.3390/jof10050366

**Published:** 2024-05-20

**Authors:** Daniela Isola, Hyun-Ju Lee, Yong-Jae Chung, Laura Zucconi, Claudia Pelosi

**Affiliations:** 1 Department of Economics, Engineering, Society and Business Organization (DEIM), University of Tuscia, Largo dell’Università Snc, 01100 Viterbo, Italy; pelosi@unitus.it; 2 Institute of Preventive Conservation for Cultural Heritage, Korea National University of Cultural Heritage, Buyeo 33115, Republic of Korea; smile2581@naver.com; 3 Department of Heritage Conservation and Restoration, Graduate School of Cultural Heritage, Korea National University of Cultural Heritage, Buyeo 33115, Republic of Korea; iamchung@nuch.ac.kr; 4 Department of Ecological and Biological Sciences (DEB), University of Tuscia, Largo dell’Università Snc, 01100 Viterbo, Italy; zucconi@unitus.it

**Keywords:** architectural wood biodeterioration, *Aspergillus niger*, cellulase activity, *Coniophora puteana*, extremotolerant fungi, FTIR, lignin degradation, metabolic plate assays, *Serpula lacrymans*, wood-decaying fungi

## Abstract

Wooden Cultural Heritage (WCH) represents a significant portion of the world’s historical and artistic heritage, consisting of immovable and movable artefacts. Despite the expertise developed since ancient times to enhance its durability, wooden artefacts are inevitably prone to degradation. Fungi play a pivotal role in the deterioration of WCH in terrestrial ecosystems, accelerating its decay and leading to alterations in color and strength. Reviewing the literature of the last 25 years, we aimed to provide a comprehensive overview of fungal diversity affecting WCH, the biochemical processes involved in wood decay, and the diagnostic tools available for fungal identification and damage evaluation. Climatic conditions influence the occurrence of fungal species in threatened WCH, characterized by a prevalence of wood-rot fungi (e.g., *Serpula lacrymans*, *Coniophora puteana*) in architectural heritage in temperate and continental climates and Ascomycota in indoor and harsh environments. More efforts are needed to address the knowledge fragmentation concerning biodiversity, the biology of the fungi involved, and succession in the degradative process, which is frequently centered solely on the main actors. Multidisciplinary collaboration among engineers, restorers, and life sciences scientists is vital for tackling the challenges posed by climate change with increased awareness. Traditional microbiology and culture collections are fundamental in laying solid foundations for a more comprehensive interpretation of big data.

## 1. Introduction

Wood has played a crucial role in humankind’s history, shaping cultures, technological advancements, and survival strategies since the earliest stages. In this context, the Clacton Spear, the oldest known worked wooden artefact dated back 400,000 years, testifies not only the strong connection of our ancestors to nature but also their vivid critical thinking and problem-solving skills. Since then, wood has been shaped to serve everyday life until the extreme journey to the afterlife. It has been forged into instruments of defense and offence, utilized to encourage mobility on land and water, and integrated as a structural component in buildings. Wood has served various purposes in human existence, including household items, ornamental objects, religious artefacts, and recreational tools. This unequivocally demonstrates that the choice of wood as one of the most used materials in mankind’s history is not solely linked to its availability and workability but also to its aesthetic qualities such as color, luster, grain, and texture [1,2].

The chemical composition of wood, along with related features, varies among species, within trees (e.g., geographic location, climate, and edaphic conditions), and across tree parts (root, stem, or branch) [3]. For this reason, people have learned, since ancient times, to carefully select wood types that best suit the intended purposes of artefacts. Often, they choose wood with inherent resistance to microbial degradation, especially for applications involving contact with the ground [4]. With technological advancements, populations learned to shield wood from microbial attacks, thereby extending the lifetime of artefacts, although this protection can lose efficacy over time. This is because wood is not merely a building material; it is a dynamic and essential ecological element that influences the health and functioning of terrestrial ecosystems. As an organic material, wood is subject to decay, playing a pivotal role in nutrient cycling and carbon storage [5,6,7].

**Figure 1:**
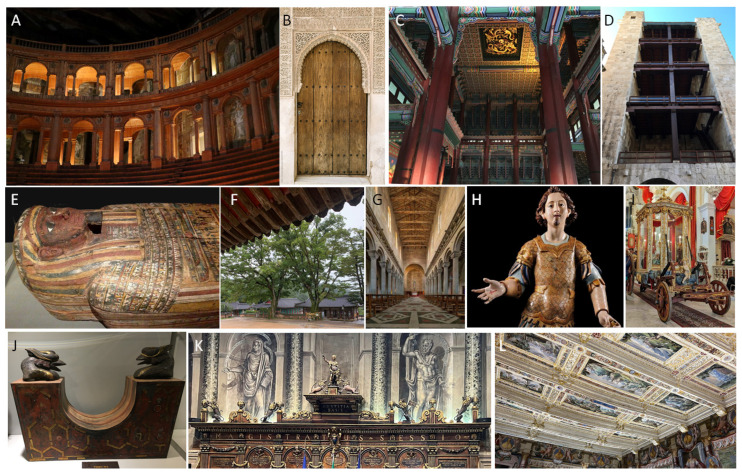
Immovable and movable terrestrial wooden cultural heritage examples. (**A**) Teatro Farnese, Parma, Italy (courtesy of Andrea Schiaretti); (**B**) gilded door, Granada, Spain; (**C**) ceiling of the Gyeongbokgung Palace, Seoul, South Korea; (**D**) Torre dell’Elefante, Cagliari, Italy; (**E**) wooden sarcophagus on display at the Egyptian Museum in Turin, Italy; (**F**) view from the Muwisa Temple, South Korea; (**G**) wooden roof of the San Lorenzo Cathedral in Viterbo, Italy; (**H**) wooden sculpture of Sant’Efisio in Cagliari, Italy; (**I**) the coach of Sant’Efisio in Cagliari, Italy (courtesy of Angelo Mocci); (**J**) wooden pillow of Queen Mureyong, Gongju National Museum, South Korea; (**K**) upper part of the wooden seat *Magistratus Sessio* in the council chamber of the Municipality of Viterbo, Italy; (**L**) Palazzo dei Priori, coffered ceiling with paintings and stuccos, Viterbo, Italy (courtesy of Emma Aronne).

Wooden Cultural Heritage (WCH) in terrestrial environments encompasses a wide range of objects. Terrestrial WCH can be roughly divided into immovable and movable artefacts. Immovable assets include structural architectural elements that can be, to some extent or entirely, exposed to external climatic conditions (e.g., historic residences and the rooftops of ancient churches) [8]. Movable WCH generally refers to indoor-stored objects, including wood panel paintings, votive sculptures, furniture, sarcophagi, and musical instruments (Figure 1). Based on the intended use of wood, it undergoes treatments aimed at improving its mechanical properties and durability. With this purpose, the isolation phase is crucial to prevent the penetration of subsequently applied materials and impregnation to prevent biological damage [9]; in the case of painted wood, ground and painted layers are also applied [10]. 

From this side, it is understandable how challenging the task is for professionals involved in the field of WCH conservation, such as wood scientists, conservators/restorers, architects, biologists, archaeologists, museum curators, and so on [11]. In this work we critically review the literature of the last 25 years, providing a comprehensive overview of: (a) wood cell structure and chemical composition, (b) fungal wood decay patterns, (c) enzymes involved in wood deterioration, with particular attention to (d) fungal diversity on WCH, (e) biological and instrumental diagnostic tools available for WCH protection, and (f) present issues and future perspectives in WCH conservation also in light of world climate change. In this way, we aim to address challenges and identify necessary improvements, providing a ready-to-use resource. 

To accomplish these goals, more than 300 peer-reviewed papers were collected using search tools from international scientific databases and other specialized sources, such as ICCROM (International Centre for the Study of the Preservation and Restoration of Cultural Property), and reports from the Korean Institute of Preventive Conservation for Cultural Property (IPCCP) and CHA (Cultural Heritage Administration).

Preparing the fungal diversity dataset, we applied some basic rules. Specifically, we updated the names of the documented taxa according to Index Fungorum (https://www.indexfungorum.org/names/Names.asp, accessed on 20 December 2023). The available accession numbers underwent reprocessing using BLASTn, and the best matches were recorded. Moreover, only one record per taxa/per location was considered, even if multiple accession numbers were available. This decision is justified as the re-isolation of the same taxon could occur when multiple samples are collected from the same location. General terms such as black fungi, yeast, *mycelia sterilia*, dematiaceous fungus, or unknown fungus were excluded because their taxonomic placement would have been uncertain or debatable. The obtained data were then analyzed and discussed.

## 2. Wood Cell Structure and Chemical Composition

To understand the nature of fungal damage and the risk to which wooden artefacts are subjected, the structure and composition of the wood should be considered. Wood (xylem) is primarily located in the trunk, branches, and roots of tree species and is formed through the activity of meristematic cells in the cribro-vascular cambium, contributing to the tree’s secondary diametrical growth. Wood composition and characteristics vary between gymnosperms, such as coniferous trees like pines and spruces, and angiosperms, also known as broad-leaved trees. Gymnosperms are characterized by an anatomically homogeneous structure, known as homoxyl wood or softwood, while angiosperms have a heterogeneous structure, known as heteroxyl wood or hardwood. 

The chemical composition of wood varies by species, among trees of the same species (considering factors like geographic location, climate, and edaphic conditions), and across different tree parts (root, trunk, or branch) [3]. Nonetheless, all of them are primarily composed of cellulose microfibrils, hemicellulose, and lignin. Cellulose, accounting for approximately 40–50% of its dry weight, provides strength and rigidity to wood fibers. Chemically, it is a linear glucose polymer linked together by β-1,4-glycosidic bonds. Conversely, hemicellulose is a branched heteropolymer composed of various sugar units. It represents approximately 20–35% of wood’s dry weight and acts as a flexible cementing matrix, holding cellulose fibers together and contributing to wood’s overall strength. Lignin, representing about 20–30% of wood’s dry weight, is the third major component. Its polymeric molecule is quite complex, composed of guaiacyl (G), syringyl (S), and p-hydroxyphenyl (H) lignin units [11,12]. Lignin binds cellulose and hemicellulose fibers together, providing structural support and conferring upon wood its hardness, resistance to decay, and brownish color. The remaining fraction, ranging between 2 and 10%, is made up of organic compounds that can be extracted from wood. The nature of these compounds can influence wood’s color, odor, and resistance to decay, serving protective and functional roles. Among these extractives, resins, tannins, oils, waxes, and various secondary metabolites are recorded [13,14,15].

**Figure 2:**
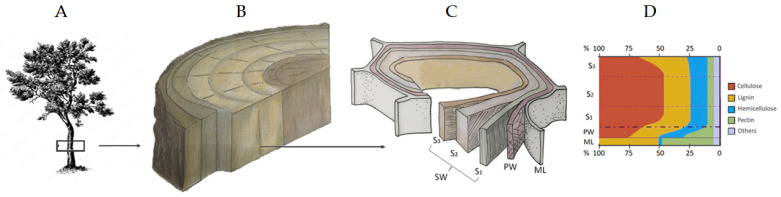
Wood, from tree to chemical composition. (**A**) tree, (**B**) trunk section, (**C**) conventional cell-wall model characterized by five cell-wall layers. The layers are the middle lamella (ML), the primary wall (PW), and the three-layer secondary wall (SW): outer (S1), middle (S2) and inner secondary wall layer (S3). (**D**) cell wall chemical composition across its different layers. The image is original; (**B**,**C**) are drawn based on [16].

The plant cell wall is a dynamic and robust structure frequently consisting of five layers where the structural compounds are differently distributed. The middle lamella (ML), the thinnest external layer holding adjacent cells together, is mainly composed of lignin and pectin. The primary wall, which is thin and elastic, consists of irregular and randomly crossing cellulose microfibrils embedded within pectin, lignin, and hemicellulose [11]. The secondary wall is usually composed of three layers (e.g., S1, S2, and S3), each differing in thickness, chemical composition, and orientation of cellulose microfibrils (Figure 2).

## 3. Fungal Wood Decay Patterns

Fungi are the primary microbial agents of wood biodeterioration in terrestrial ecosystems and are traditionally grouped based on the macroscopic and microscopic deterioration patterns they produce. Color, texture, and chemical changes are used to discriminate among brown rot (BR), white rot (WR), and soft rot (SR) fungi [17,18]. The variations in wood degradation patterns can be attributed to local fluctuations in the microdistribution of chemical components within wood, including lignin, cellulose, and hemicelluloses. Other influencing factors include the presence of extractives and the consequences of wood treatments, such as preservatives or chemical modifications [19,20].

**Figure 3:**
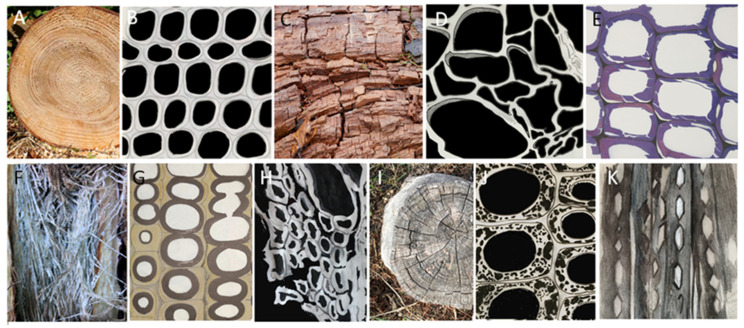
Macroscopic wood appearance and drawings illustrating healthy and rotted wood patterns as can be observed under a microscope. (**A**,**B**) Healthy wood: (**B**) intact tracheid cell walls (drawn based on [45]). (**C**–**E**) BR decay: (**C**) wood cubical pattern; (**D**) The degradation of cellulose in woody cell walls leaves a residual network of lignin. Cell walls collapse and appear distorted (drawn based on [5]); (**E**) numerous splits in the secondary walls of tracheids (drawn based on [46]). (**F**–**H**) WR decay: (**F**) white stringy rot; (**G**) simultaneous white rot – in the dark areas lignin, cellulose and hemicellulose are degraded approximately at the same rate starting from the lumina (drawn based on [39]); (**H**) preferential white rot, lignin in secondary walls, and ML is selectively degraded while the rest of the cellulose rich cell wall is maintained and cells result separated from the adjacent (drawn based on [47]). (**I**–**K**) SR decay: (**I**) soft rotted wood; (**J**) SR type I, the fungal infection caused many cavities inside the wood cell wall especially in the S2 layer (drawn based on [45]); (**K**) SR type I, chains of diamond-shaped cavities extend longitudinally through the S2 cell wall layer (drawn based on [48]).

BR decay was named after the brown discoloration of wood resulting from the degradation of all wood carbohydrates, including crystalline cellulose. Following this process, a residual chemically modified lignin matrix remains and undergoes gradual conversion into humic substances through long-term interactions with other microorganisms [21,22]. Brown rotted wood becomes brittle and exhibits a typical cross-crack in a cubical pattern when dry [5] (Figure 3C). This damage is mediated by both enzymatic and non-enzymatic mechanisms, selectively removing wood polysaccharides [20,23]. Wood infection begins with the penetration of cell lumina and the release of enzymes such as cellulase, mannanase, and xylanase, along with non-enzymatic compounds like hydrogen peroxide, Fenton reagent (H_2_O_2_/Fe^2+^, [24], and oxalic acid when the hyphae are in proximity to the S3 cell wall layer. These substances collectively initiate the degradation of the S2 layer [25,26]. In the early stages, the cells do not appear altered; later, they lose rigidity, becoming porous, and the lignin residuals collapse (Figure 3D), or splits and cracks develop in the secondary wall (Figure 3E). The middle lamella (ML) remains intact until the late stages of decay.

Phylogenetic analyses evidenced as brown rot fungi evolved from a saprobic white rot ancestor progressively losing energetically expensive white rot mechanisms of ligninolysis (e.g., peroxidases, laccases) and reducing genes encoding carbohydrate-active enzymes (CAZymes) in favor of a refined suite of decay genes typical of brown rot [27,28]. Many BR fungi show a preference for coniferous timber belonging to the Pinaceae family [29,30]. This preference has been attributed to the different chemistry characterizing softwoods and hardwoods. However, recent evolutionary studies based on a broader dataset have shown that most brown rot fungi (all belonging to the Basidiomycota phylum) are generalists. Only two of the five brown rot clades, namely the *Gloeophyllum*-*Neolentinus* and *Serpula*-*Hygrophoropsis* clades, primarily display gymnosperm specialists [31]. Examples of brown rotting fungi include *Antrodia sinuosa* (Fr.) P. Karst., *Coniophora puteana* (Schumach.) P. Karst., *Fibroporia vaillantii* (DC.) Parmastro, *Gloeophyllum sepiarium* (Wulfen) P. Karst., *Schizophyllum commune* Fr., and *Serpula lacrymans* (Wulfen) P. Karst.

WR decay is named after the bleaching of normal wood coloration due to the degradation of all cell wall components, including lignin. Other discoloration ranging from yellow to violet or red could happen due to lignin oxidation [23,29]. Furthermore, white rotted wood also appears soft and spongy because of the created void spaces or shows a fibrous texture separating into string-like fragments [23,32]. Consequently, white rotted wood adsorbs a lot of water or feels light and soft when dry [11,29]. Wood degradation is mainly performed through enzymatic processes and involves all the structural components, even the more recalcitrant lignin and crystalline cellulose. The degradation dynamic of lignin allows us to distinguish between two white rot decay patterns: simultaneous rot and preferential rot. In simultaneous white rot, all major structural chemicals are eroded at the same rate from the lumen to the ML, resulting in a uniform and gradual loss of cell wall thickness [32] (Figure 3G). Conversely, in preferential rot, lignin is selectively degraded before the degradation of cellulose and hemicelluloses [32]. Initially, lignin removal can be observed in the S3 and S2 layers near the hyphae, leaving the cellulose fibrils exposed and proceeding along the circumference. Subsequently, lignin degradation also involves the S1 layer down to the ML, resulting in a granular appearance [32]. The typical deterioration pattern of preferential white rot (Figure 3H) is characterized by a relatively conserved cell wall and the loss of the middle lamella, which could eventually be maintained in the corners [19]. White rot damage is mainly caused by Basidiomycota especially by members of the order Polyporales [23]. From an evolutionary point of view, it has been suggested that white rot derives from an ancestral soft rot decay machinery conserved across Asco- and Basidiomycota [33,34]. Examples of fungi leading to simultaneous white rot include *Fomes fomentarius* (L.) Fr., *Trametes versicolor* (L.) Lloyd, and *Phlebia radiata* Fr. [23], while *Porodaedalea pini* (Brot.) Murrill (syn. *Trametes pini*), *Stereum hirsutum* (Willd.) Pers., *Inocutis dryophila* (Berk.) Fiasson & Niemelä (syn. *Inonotus dryophillus*), and *Bjerkandera adusta* (Willd.) P. Karst are associated to preferential white rot [23,35]. Several examples of white rot fungi capable of inducing both degradation patterns in the same substrate have been notably recorded. In his research on fungal degradation of wood cell walls Daniel explained this phenomenon in relation to the microenvironmental conditions [20].

SR decay derives its name from the spongy soft texture developed on the surface of damaged waterlogged wood [36]. In terrestrial environments, instead, soft rotted wood turns gray and even black in late stages. The surface tends to crack, exhibiting the typical brash fractures, leading to a significant reduction in its strength [36]. The term ‘soft rot’ was nevertheless retained to distinguish the wood decay caused by ascomycetes from rots caused by the wood-destroying basidiomycetes [35]. Cellulose and hemicellulose are the main structural components decomposed by soft rot fungi while lignin is slightly modified. Depending on the alteration caused to wood cell walls, morphologically different decay patterns are known as Type I (cavity formation) and Type II (cell wall erosion) [37,38,39,40,41]. Type I attack is characterized by chains of conical shaped cavities that follow the microfibrillar structure producing a spiral of minute holes (sometimes biconical with rhomboid or diamond shape) within the secondary wall [5,38,40], appreciable in longitudinal sections (Figure 3J). In transverse sections, numerous holes can be recorded especially in the S2 layer (Figure 3K). Type II soft rot is a general erosion of the wood cell wall layers starting from the S3-lumen interface and working outward with no involvement of the middle lamella [38,42]. Despite this categorization, it is not rare that both Type I and Type II attacks can be produced by the same fungus in the same sample [42]. Several Ascomycetous fungi have been associated with the soft rot decay pattern, among them *Cadophora malorum* (Kidd & Beaumont) W. Gams and *Chaetomium globosum* Kunze.

**Figure 4:**
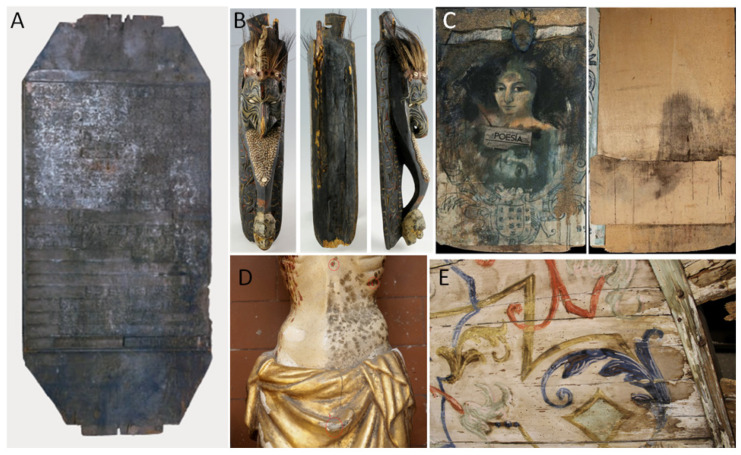
Examples of macroscopic wooden artefact alterations. (**A**) Temple woodblock affected by white rot; (**B**) 19th-century polychrome wood mask; (**C**) *recto* and *verso* of Poesia by Kokocinski, a composite artwork (paper and paint on multi-layered wooden panel) affected by fungal colonization. (**D**) Discolored painted layer of a votive statue representing the crucifixion; (**E**) biological attack on the wooden ceiling of the Palazzo Tarquini-Savelli, Marta (VT), Italy.

Another wood alteration involves pigmented fungi leading to permanent discoloration. Such color alteration is known as sapstain, or bluestain, as the most common colorations are black, grey, brown, or blue, with each color showing different intensities depending on the fungi responsible for the stain (Figure 4). Green, yellow, pink, and reddish discolorations have also been recorded [23]. While rot fungi feed on structural components such as lignin, cellulose, and hemicellulose, staining fungi act on free sugars, nutrients, and wood extractives [11,20,43]. Hundreds of fungi can cause wood discoloration, with the majority falling into three main groups: *Ophiostoma*/*Ceratocystis*, black yeasts, and black molds [18,23]. Different to ophiostomatoid fungi, black yeasts and dark moulds are generally regarded as surface stainers. *Ceratocystis imperfecta* (V.V. Mill. & Tcherntz.) C. Moreau, *Ceratocystis pilifera* (Fr.) C. Moreau (syn. *Ophiostoma piliferum*), *Ceratocystis minor* (Hedgc.) J. Hunt (syn. *Ophiostoma minus*), *Ceratocystis piceae* (Münch) B.K. Bakshi (syn. *Ophiostoma piceae*), as well as *Aureobasidium pullulans* (de Bary & Löwenthal) G. Arnaud, *Sydowia polyspora* (Bref.) E. Müll., *Cladosporium* spp. and *Alternaria* spp. have been reported as frequent agents of sapstain [44]. 

## 4. Enzymes Involved in Wood Degradation

### 4.1. Polysaccharides Degradation

The degradation of cellulose is an important process involving the hydrolysis of β-1,4-glycosidic bonds. This prerogative is common in plant pathogens and is mediated by the cellulase enzyme complex, also known as cellulases, which is a subset of the Carbohydrate-Active Enzymes (CAZymes). A significant category of enzymes involved in the degradation of cellulose is called glycoside hydrolases (GH) in which we can classically distinguish three main groups: (i) endoglucanases (EGs), (ii) cellobiohydrolases (CBHs), and (iii) betaglucosidases (BGLs) [49,50,51]. EGs cut at unpredictable locations within the insoluble crystalline cellulose polysaccharide chain, yielding oligosaccharides of varying lengths generating new chain ends and creating an open site for the CBHs that cleaves cellulose to release cellobiose or cellulo-oligosaccharides. The last enzyme BGLs has the function of degrading cellobiose into glucose [52]. Cellulose degradation involves other enzymes such as polysaccharide monooxygenases (PMOs), copper-containing oxidases that work synergistically with other enzymes [53]. Hemicelluloses, instead, are initially targeted by endo-enzymes such as mannanases and xylanases producing shorter chains that are subsequently hydrolysed into simple sugars by glycosidases (e.g., mannosidases and xylosidases) [11].

### 4.2. Lignin Degradation

Due to the highly variable lignin polymeric structure and the diversity of lignin unit bonds, the extracellular enzymes produced by fungi are rather diversified and proceed by less specific oxidative mechanisms [54]. As a result, lignin cannot be easily utilized as a carbon or energy source. Its biological degradation is primarily aimed at making cellulose and hemicellulose accessible for further breakdown. Efficient biocatalysts, known as ligninases, play a crucial role in the oxidation of lignin, utilizing strong oxidants like H_2_O_2_ and O_2_ as electron acceptors. Ligninases (also known as lignin-modifying enzymes, LME) can be broadly categorized into heme peroxidases and laccases. An additional group includes auxiliary enzymes responsible for hydrogen peroxide production [32,42,54,55,56]. 

#### 4.2.1. Heme Peroxidases

Lignin oxidation requires effective biocatalysts that employ potent oxidants as electron acceptors, such as H_2_O_2_ and O_2_. Several heme peroxidases play a pivotal role in the degradation of wood, and include lignin peroxidases, manganese peroxidase, versatile peroxidases, and dye-decolorizing peroxidases.

Lignin peroxidases (LiPs) oxidize both phenolic and nonphenolic lignin groups at the lumen surface (S3 layer). The proposed mechanism involves the oxidation of smaller intermediates, such as veratryl alcohol, whose radical size allows it to erode the wood cell wall from the lumen surface and potentially participating to its progression outward.

Manganese peroxidases (MnPs) are extracellular glycosylated enzymes containing one ferric protoheme IX per molecule. A distinctive feature of MnPs is their utilization of Mn(II) as the reducing substrate, producing Mn(III) that permeates the lignocellulose structure and subsequently oxidizes diverse monomeric phenols. Glutathione and other mediators are required for the degradation of high redox potential nonphenolic compounds [57].

Versatile peroxidases (VP) are considered a hybrid between LiPs and MnPs. Unlike other lignin-degrading peroxidases, VPs can directly oxidize high redox potential phenolic and nonphenolic substrates without the need for mediators, making them versatile in lignin breakdown [32,58]. 

Dye-decolorizing peroxidases (DyPs), like VPs, use hydrogen peroxide as an electron acceptor. They can oxidize Reactive Black 5 (azo dye), phenols, and veratryl alcohol, but unlike VPs they also have the ability to oxidize recalcitrant anthraquinone dyes [59,60,61].

#### 4.2.2. Laccases

Laccases belong to the broader class of phenol oxidases, which is a group of enzymes responsible for catalyzing the oxidation of phenolic compounds, including tyrosinases [62]. Laccases are multi-copper oxidases basically containing four copper atoms. One of these copper atoms determines the substrates to be oxidized according to its redox potential, while the three other copper atoms transfer the electrons to O_2_ that is then reduced to water. Laccases catalyze the one-electron oxidation of a wide range of compounds including di- substituted phenols and polyphenols, and di- and aromatic amines to form free radicals, which in turn can non-enzymatically produce dimers, oligomers, and polymers [49]. 

#### 4.2.3. Lignin-Degrading Auxiliary Enzymes (LDA)

These enzymes, while incapable of independently breaking down lignin, are necessary for the overall degradation process. Auxiliary enzymes facilitate the sequential action of multiple proteins, which can result in the oxidative generation of hydrogen peroxide required by all four heme peroxidases [39,56]. This group includes, for example, glyoxal oxidase, aryl alcohol oxidases, cellobiose dehydrogenase, and glucose oxidase [56].

#### 4.2.4. Auxiliary Non-Proteinaceous Molecules

In the early stages of decay, a variety of oxidative and hydrolytic enzymes produced by white rot fungi cannot penetrate sound wood cell walls. In this stage of the depolymerization process, the involvement of low molecular weight redox mediators, such as veratryl alcohol, fatty acids, and Mn(II) has been proved [63]. While oxalic acid plays an important role in decreasing pH to the optimal level for the proper functioning of ligninolytic enzymes cut, also acting as an electron donor in lignocellulose degradation [64].

## 5. Fungal Diversity on WCH

### 5.1. Geographic Distribution

**Figure 5:**
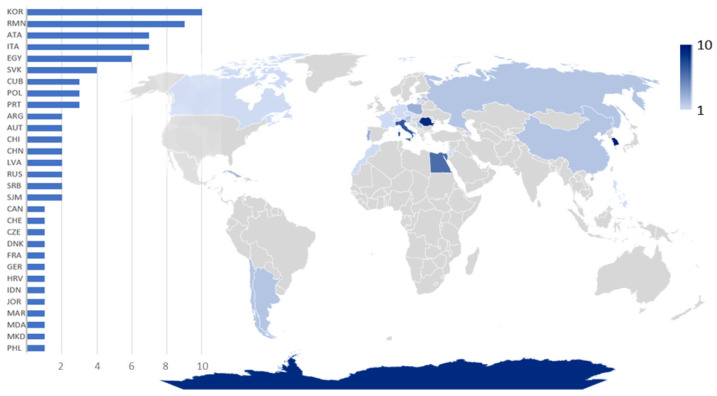
Geographical distribution of the wooden cultural heritage studied in the 81 papers for which mycological investigations were performed. The countries are indicated with the international three letters alpha-3 code: KOR: South Korea, RMN: Romania, ATA: Antarctica; ITA: Italy, EGY: Egypt, SVK: Slovakia, CUB: Cuba, POL: Poland, PRT: Portugal, ARG: Argentina, AUT: Austria, CHI: Chile, CHN: China, LVA: Latvia, RUS: Russia, SRB: Serbia, SJM: Svalbard, CAN: Canada, CHE: Switzerland, CZE: Czechia, DNK: Denmark (Greenland), FRA: France, GER: Germany, HRV: Croatia, IDN: Indonesia, JOR: Jordan, MAR: Morocco, MDA: Moldova, MKD: North Macedonia, PHL: Philippines.

Only a limited fraction of the scientific papers produced in the last 25 years focus on degradation caused by fungi in terrestrial environments. In fact, only 81 papers provided taxonomic information regarding fungal deteriogens. The research outcomes were heterogeneous. While certain countries exhibited a pronounced interest in fungi linked to the decay of wooden cultural heritage, as evidenced by detailed studies on Korean printing wood blocks, Romanian and Chilean wooden churches, and the architectural heritage of Antarctic expeditions (Figure 5), others, such as some Italian investigations, lacked comprehensive investigations into fungal diversity, often reporting only a single fungal taxon.

**Figure 6:**
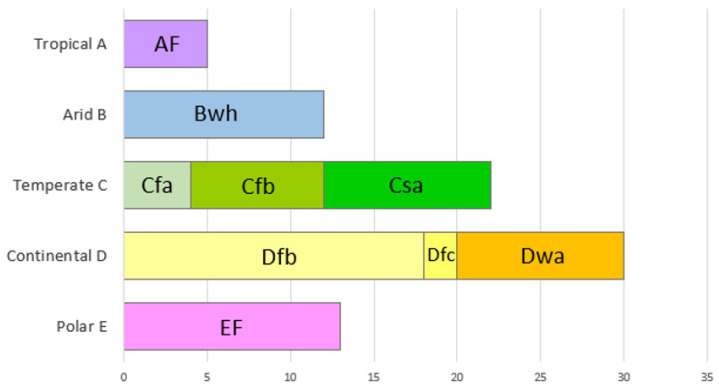
Papers sorted by Köppen–Geiger climatic regions. AF: tropical rainforest climate; Bwh: hot desert climate; Cfa: humid subtropical climate; Cfb: temperate oceanic climate or subtropical highland climate; Csa: hot-summer Mediterranean climate; Dfb: warm-summer humid continental climate; Dfc: subarctic climate; Dwa: humid continental climate; EF: ice cap climate. The *x*-axis indicates the nr of papers.

Additional insights could be gained by sorting papers based on climatic regions. In this perspective the Köppen–Geiger climate classification represents a highly suitable tool to aggregate complex climate gradients into a simple but ecologically meaningful classification scheme [65]. For this reason, it is often used when analyzing the distribution of species or setting up dynamic global vegetation models. This classification is based on temperature and precipitation patterns and comprises major climate types, such as tropical—A, arid/dry—B, temperate—C, continental—D, and polar—E [65]. As shown in Figure 6, further sub-categories were present. AF: Tropical rainforest climate (Cuba, Indonesia, and the Philippines), Bwh: hot desert climate (Argentina, Egypt, Jordan, and Morocco), Cfa: humid subtropical climate (China, Croatia, and North Macedonia), Cfb: temperate oceanic climate or subtropical highland climate (Czechia, France, Germany, Serbia), Csa: hot-summer Mediterranean climate (Italy and Portugal), Dfb: warm-summer humid continental climate (Austria, Latvia, Moldova, Poland, Romania, Slovakia, and Switzerland), Dfc: subarctic or subpolar climate (Russia), Dwa: humid continental climate (South Korea), EF: ice cap climate (Antarctica, Canada, Greenland, and Svalbard). The climatic sub-category attribution has been determined based on the locations where the surveys were conducted. A higher number of papers has been recorded from the countries characterized by continental climate (37%), with a prevalence of the Dfb sub-category (22.2%) defined by warm-summer humid continental climate.

### 5.2. Fungal Diversity

A comprehensive dataset was created for the fungal taxa associated with deteriorated wooden artefacts of historical or artistic interest (Table 1). Even though some of the reviewed papers contained information about the so-called ‘slime moulds’ and lichens (e.g., [66,67]), they were not included, limiting our focus to the three major divisions in the kingdom of Fungi, namely Ascomycota, Basidiomycota, and Mucoromycota [68]. In total, 612 taxa were recorded, representing 472 identified species (77%), and 105 (17%), 23 (3.75%), and 12 (1.9%) strains identified at genus, family, and order levels, respectively.

**Figure 7:**
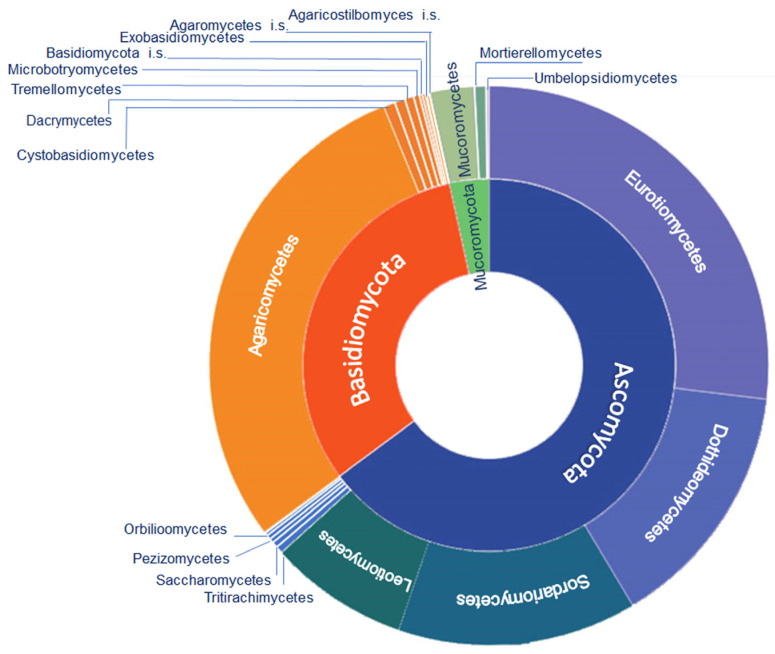
High taxonomic rank distribution of the 1167 fungal records found on wooden cultural heritage artefacts. Division (central ring) and class (outer ring). Different shades of the same color indicate the classes belonging to the same division.

From a taxonomical viewpoint (Figure 7), a higher prevalence of Ascomycota (63.8%) has been recorded compared to Basidiomycota (32.6%) and Mucoromycota (3.6%). Interestingly, 90% of Basidiomycota records belong to Agaricomycetes, while the identified Ascomycota are mainly distributed within Eurotiomycetes (42%), Dothideomycetes (22.4%), Sordariomycetes (21.4%), and Leotiomycetes (12.3%). Approximately 70% of Mucoromycota is represented by Mucoromycetes with a dominance of the genus *Rhizopus* (31%). 

**Figure 8:**
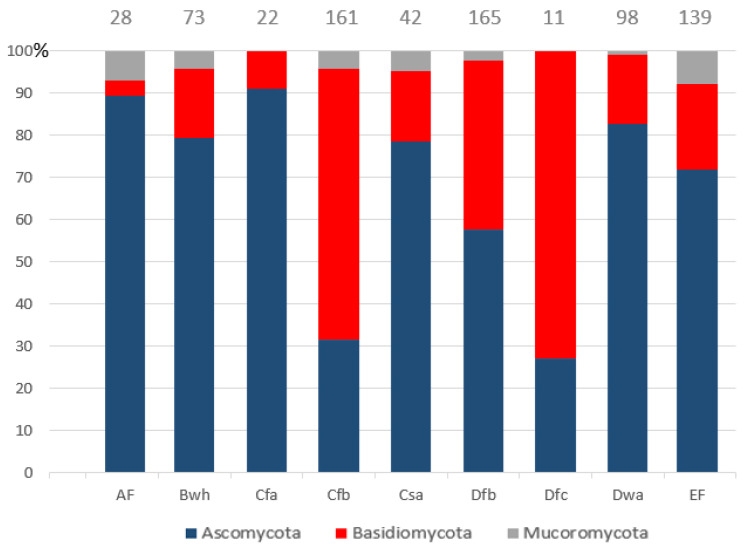
Prevalence of phyla sorted by climatic sub-areas. Values are presented as a percentage of the total taxa recorded (indicated above each column) for each climatic sub-area. Ascomycota is represented in blue, Basidiomycota in red, and Mucoromycota in grey. AF: tropical rainforest climate; Bwh: hot desert climate; Cfa: humid subtropical climate; Cfb: temperate oceanic climate or subtropical highland climate; Csa: hot-summer Mediterranean climate; Dfb: warm-summer humid continental climate; Dfc: subarctic climate; Dwa: humid continental climate; EF: ice cap climate.

The frequency of fungal divisions locally changes when data are ordered by climatic sub-areas (Figure 8). In detail, a prevalence of Basidiomycota has been recorded in temperate oceanic (Cfb) and subarctic climate (Dfc). Ascomycota values above 70% are recorded in polar (EF), hot desert (Bwh), humid subtropical/highland (Cfa), Mediterranean (Csa), humid continental (Dwa) and tropical rainforest (AF) climates. Nevertheless, these data should be weighted on the base of the number of taxa isolated per area.

**Figure 9:**
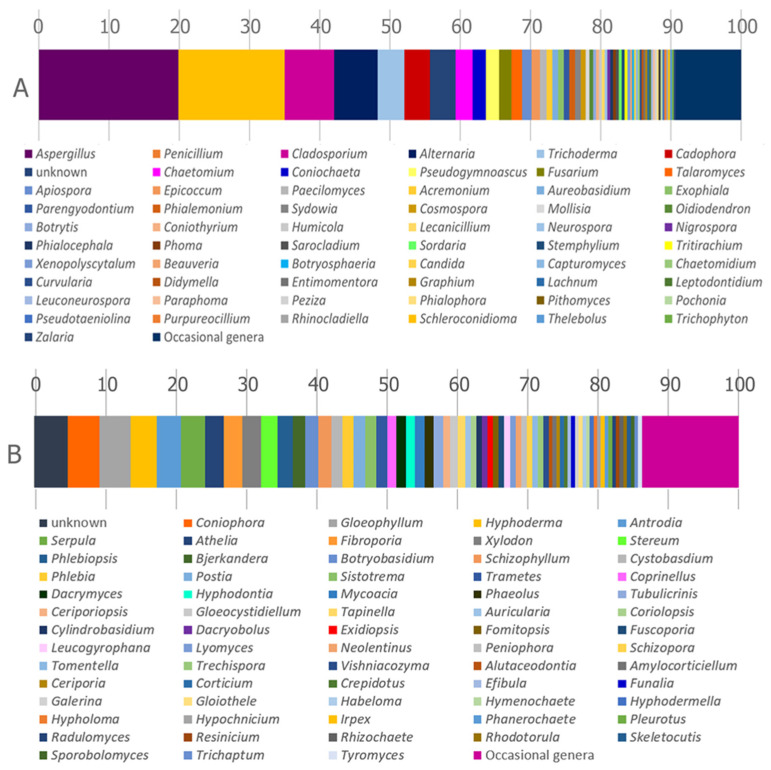
Wooden cultural heritage genera frequency based on the 1167 fungal records. (**A**) Ascomycota, (**B**) Basidiomycota. To improve reading, the genera found have been ordered by frequency, while those found only once have been merged and indicated as “Occasional genera”. Values are expressed as percentages.

As reported in Figure 9A, the 52% of the Ascomycota records is represented by five genera: *Aspergillus* (19.86%), *Penicillium* (15.13%), *Cladosporium* (7.02%), *Alternaria* (6.21%), and *Trichoderma* (3.78%). Besides, the five most frequent basidiomycetous fungi, accounting for 19.7% of the total Basidiomycota records, are *Coniophora* (4.5%), *Gloeophyllum* (4.5%), *Hyphoderma* (3.7%), *Antrodia* (3.5%), and *Serpula* (3.5%) respectively (Figure 9B). 

**Figure 10:**
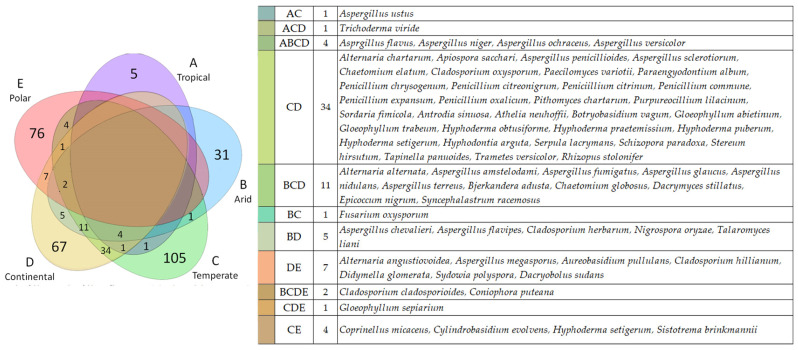
Venn diagram depicting fungal species sorted by the main climatic groups.

Of the 472 isolated species, the majority (60%) are exclusive of the climatic areas in which they were isolated (Figure 10). The percentage of shared species ranges from 15.6% recorded for Polar regions (E) to 54% as recorded for hot tropical climates (A). The highest number of shared species (34) was recorded between temperate (C) and continental (D) climate areas. Among them were species belonging to all the phyla considered, such as *Penicillium chrysogenum*, *Aspergillus penicillioides*, *Serpula lacrymans*, *Trametes versicolor*, and *Rhizopus stolonifer*. Broader distribution within the climatic areas was recorded for *Gloeophillum sepiarium* (CDE, temperate/continental/polar), *Trichoderma viride* (ACD, tropical/temperate/continental), and 11 species are shared by arid/temperate/continental (BCD); among them *Alternaria alternata*, *Aspergillus fumigatus*, *Aspergillus terreus*, *Bjerkandera adusta*, *Chaetomium globosus*, and *Epicoccum nigrum*. Additionally, *Cladosporium cladosporioides* and *Coniphora puteana* were recorded from WCH originating from all climatic areas except the tropics (i.e., BCDE); while four *Aspergillus* species (namely *A. flavus*, *A. niger*, *A. ochraceus*, *A. versicolor*) were recorded worldwide except in polar WCH (ABCD). 

### 5.3. Environmental Factors Affecting Fungal Growth

Water is a basic element for the conservation of the physical mechanical features of wood [146], but it is also the most common limiting factor in terrestrial ecosystems, able to shape life forms and biodeteriorative processes according to Liebig’s laws [147]. Moisture and temperature have been proven to be crucial factors in the growth of fungi and the decomposition of wood, both indoors and outdoors [18,148,149]. 

Indeed, water provides the necessary environment for fungal growth, enabling hyphal penetration into wood structures and also allowing extracellular transport of fungal metabolites [18,150]. Moisture activates enzymatic processes in fungi, but it is also essential in non-enzymatic reactions involving hydrogen peroxide influencing the overall decay process [24]. Wood is a porous material that can contain and/or acquire water in both liquid and gaseous form [18]. From this side, we can distinguish different parameters such as wood moisture content (MC), representing the water fraction with respect of the wood weight (expressed as percentage), a fiber saturation point (FSP, ~28–30%), relative humidity (RH) indicating the water content in the air (expressed as percentage), and water activity (a_w_), namely the water available for biological/fungal growth [151]. While a_w_ is generally used, MC is employed for wood rot fungi (Table 2).

The constraints that temperature imposes on vital phenomena are primarily linked to its effects on the physicochemical properties of water and its changes in state. Temperature could change the RH and water content of materials (e.g., water condensation on cold surfaces) but also affect chemical reactions kinetics and, as a consequence, fungal metabolism and growth rates. Oxygen is required for fungal decaying processes, and the minimum air volume in wood for degradation is between 10 and 20% [148].

The influence of wood MC and temperature are the objects of intensive studies aimed at quantifying the risk of fungal decay and predicting the service life of wooden objects [152]. From this perspective, there is a critical need to define a minimum moisture threshold (MMThr) necessary for the onset and subsequent progression of wood decay. Additionally, defining optimum and maximum MC values is essential to fully understand the growth and decay conditions for basidiomycetes [149]. 

Table 2 illustrates the variability in water and temperature requirements for fungal growth. With a few exceptions, the most common temperature optimum is between 20 and 25 °C. Significant variations are noticeable in water requirements, with many xerotolerant ascomycota with a_w_ values below 0.80, when most wood-destroying basidiomycetes are hydrophilic with a_w_ 0.97 [153]. This strong affinity for water becomes evident through the progressive increase in moisture content (MC) values during the degradative process. It is acknowledged that *Serpula lacrymans* can draw large amounts of water over considerable distances with its mycelial cords. 

These growth parameters have been employed to establish guidelines such as ASHRAE 160 [154] for moisture control in buildings and EN 335 [155] for wood conservation. The latter sets a crucial threshold for wood moisture content (MC) below 20%, essential for reducing the risk of fungal growth and deterring insect infestations. However, these values may undergo slight variations depending on the type of wood or processing method used in artefact making [18]. Indeed, wood frequently served as a support for further decoration in WCH. Therefore, it is of utmost importance to evaluate the artefact in its entirety in relation to the surrounding environment (indoor/outdoor, controlled/not controlled indoor conditions). If from one side excessively damp wood is susceptible to decay and fungal growth, an overly dry wooden artefact may become brittle and prone to cracking [156]. 

Precipitation patterns and air temperature significantly influence wood decay rates, with the duration of rainfall (constant conditions) being more critical than the total amount. Maintaining stable moisture and temperature conditions is crucial for wood decay [153,157]. In heated buildings, the wood moisture content ranges from 6% to 15%, making it generally too dry for fungi, while 45% could be recorded in winter nights by condensation with the potential for higher levels due to construction features and occupants’ practices [158].

**Table 2:** Water and temperature requirements for fungal growth. For Basidiomycetes, MC is expressed as a percentage. Water activity (a_w_) scale range from 0 to 1.

Fungal Species	Temperature °C			a_w_	Wood Moisture Content MC			References
	Min	Opt	Max		Min	Opt	Max	
*Alternaria alternata*		21		~0.85–0.89				[159]
*Aspergillus flavus*	18	30	45	~0.78–0.84				[150]
*Aspergillus fumigatus*	12	37–43	57	~0.82–0.85				[160]
*Aspergillus niger*	11–13		47–48	~0.78–0.77				[160]
*Aspergillus terreus*	11	40	48	~0.78				[161,162]
*Aspergillus versicolor*	4	21–22	40	~0.74–0.79				[160]
*Aureobasidium pullulans*	2	25	35	~0.89–0.90				[23]
*Cadophora fastigiata*	0	15	35					[160]
*Cadophora malorum*	5	24	30					[160]
*Chaetomium globosum*	4–10	16–25	38	~0.94				[160]
*Cladosporium cladosporioides*	−10	20	35	~0.85–0.87				[163]
*Cladosporium herbarum*	−10	20	35	~0.85				[163]
*Cladosporium sphaerospermum*	2	15		~0.82–0.85				[160]
*Epicoccum nigrum*	−3	23–28	45	~0.97–0.99				[160]
*Penicillium brevicompactum*	−2	23	30	~0.75–0.79				[164]
*Penicillium chrysogenum*	3	24	36	~0.78–0.85				[150]
*Penicillium expansum*		23–26		~0.80–0.84				[164]
*Pseudogymnoascus pannorum*	0	15	28	~0.89				[160,165]
*Trichoderma viride*		25–30		~0.90–0.95				[166,167]
*Anthrodia sinuosa*		25–30	35		30	35–55	60–90	[23]
*Anthrodia xantha*	5	25–30	35		30	35–55	60–90	[23]
*Coniophora puteana*	5	22.5–25	40		21.9–29.7	30–70	60–80	[23,168,169]
*Fibroporia vaillantii*	3	28	36		40–50			[23]
*Gloeophyllum abietinum*					25.0–30.1	55.4–125.2		[158]
*Gloeophyllum sepiarium*	5	26–35	46		30		60	[170]
*Schizophyllum commune*		28–36	44					[23]
*Serpula lacrymans*	5	20	25	~0.95–0.993	26	30–60	55–225	[171,172]
*Sistotrema brinkmannii*	5	25	30					[173]
*Trametes versicolor*		24–33	34–40		15.4–16.3			[23,158,169]
*Rhizopus stolonifer*	10	26	36					[161]

Notably, even in meticulously controlled environments such as a museum repository, fungal colonies were unexpectedly detected on wooden sculptures and paintings [120]. Furthermore, the detection of cold-tolerant and endemic fungi demonstrates that these fungi continue to grow on objects even when stored between 8 °C and 10 °C [133]. Poor ventilation, promoting the persistence of elevated levels of RH, is considered a contributing factor for fungal outbreaks [148].

## 6. Biological and Instrumental Diagnostic Tools

Diagnostic tools play a fundamental role in preserving cultural heritage. Specifically, they allow for providing essential answers. While biological diagnostics resolve questions about ‘who’ caused the damage and ‘why’ they did it, instrumental diagnostics define ‘what’ the conservation status of the artwork is, ‘how’ it was constructed, and the extent of the damage that has occurred.

### 6.1. Biological Tools

#### 6.1.1. Identification Methods 

Although morphological identification is accepted and acceptable in many contexts, in recent decades, with the advent of molecular techniques, we have witnessed significant changes in fungal taxonomy. These changes have included notable exclusions from the fungal kingdom, the cessation of using different names for the sexual and asexual phases of pleomorphic species, and the description of new species recognizable only through molecular methods [174,175]. 

The changes that occurred reflected both technical challenges, such as the difficulty of identifying wood-rotting fungi from cultures or having reference sequences in databases [176,177,178] and the advancements in molecular techniques with a gradual shift from morphological to molecular identification and from culture-based to high-throughput sequencing. In parallel with these advancements, researchers have explored alternative methods for fungal identification, for example MALDI-TOF [79,179,180] and FTIR [176,181]. 

The majority of the 81 studies reporting data on fungal diversity associated with terrestrial WCH indicated a dominance of culture-based methods, associated with both morphological (25.9%) and molecular identification (55.6%). Another substantial portion (17.3%), utilizes instead direct morphological identification. These data are even more interesting as over 70% of these studies were focused on wood-rotting basidiomycota from architectural WCH (e.g., [66,135,137,141,142]). Culture-independent methods, on the other hand, were used in less than 2% of the selected papers.

The culture media used varied depending on the research purposes and environment from which samples were taken. Standard media such as potato dextrose agar (PDA) [182], Sabouraud dextrose agar (SDA) [183], and Czapek-Dox (Cz) [184], were frequently used. Notably, recipes using malt extract were rather diversified and comprised malt agar (MA, malt extract 30 g, agar 15 g/L) [185], malt extract agar (MEA; maltose 12.75 g/L, dextrin 2.75 g/L, glycerol 2.35 g/L, peptone 0.78 g/L, Agar 15 g/L) [186], and medium EM (malt extract 40 g, agar 20 g/L) to different MEA dilutions (1.5% or 2%), or were furtherly acidified with citric acid or lactic acid [45,71,123]. Other standard media such as DRBC (Dichloran Rose Bengal Chloramfenicol Agar) [187], Cook’s Rose Bengal (CRB) [188], Yeast dextrose agar (YDA; dextrose, 10 g, yeast extract, 10 g, Agar, 15 g) [189] were used. In addition, very selective media were used such as Cellulose Agar (Cell-A) and Lignin Agar (Lignin-A) [75,102], or those characterized by low a_w_ to isolate xerotolerant and/or xerophilic fungi such as DG18 (dichloran 18% glycerol agar), SA (Salt Agar; 0.1% (w/v) malt extract, 0.67% (w/v) nitrogen base, 5% (w/v) glucose, 2% (w/v) agar, with the addition of 10% (w/v) NaCl), and MEA supplemented with 7.5% NaCl [90,96,111]. A semi-selective medium for basidiomycota was also used (malt extract 15 g, agar 15 g, yeast extract 2 g, benlate 0.06 g, streptomycin sulfate 0.01 g, and 2 mL of lactic acid) [45,71,123].

Many research groups employed up to six different culture media to increase the probability of isolation. In contrast, approximately 46% of studies based on a culturing approach utilized only one medium and one temperature for incubation, mostly within the range of 25–30 °C. Moreover, about 77% of culture-based studies applied relatively short incubation times (up to 7 days) or did not provide any information about it. Only studies focusing on polar WCH regularly used a medium for basidiomycota. These data indicate ongoing opportunities to enhance the isolation of fungi from WCH. When evaluating the protocol’s costs and benefits, it is important to remember that rapid results may lack comprehensiveness. Although standard culture media expedite preparation and growth observation, the prevalence of simple sugars and optimal mesophilic temperatures can boost competitive fungi, masking other taxa involved in biodeterioration. To gain a deeper insight into the deteriorative process, utilizing diverse culture media, varied incubation temperatures, and extended incubation periods are necessary.

#### 6.1.2. Detrimental Potential

The metabolic capacity to degrade the structural elements of wood like cellulose, hemicellulose, and lignin is crucial in determining the detrimental potential of a fungal species for WCH. Basidiomycetes, acknowledged as “wood rotting fungi”, are swiftly classified as white rot or brown rot once identified, given the substantial significance of wood and the economic harm linked to them. This categorization immediately offers insights into the types of enzymes they can produce—cellulase, hemicellulase, or ligninase—and, consequently, the potential risks for the material [23]. For filamentous ascomycetes and yeasts (both asco- and basidio-mycetes), often considered as having minimal impact on structural wood degradation, their metabolic capabilities and associated risks for WCH may not be readily perceived. 

Currently, plate assays remain an efficient and cost-effective method for profiling the detrimental potential of microorganisms based on their degradation abilities [190]. Furthermore, they serve as a cleaver tool to distinguish organisms with a prominent role in decay from others within the community colonizing an artefact.

Microorganisms producing cellulase can be screened, utilizing a cellulosic substrate like filter paper, microcrystalline cellulose (e.g., Avicel), or carboxymethylcellulose (CMC) as the sole carbon source for their growth [191], or less frequently in association with simple sugars (e.g., [83,192]). The use of filter paper, insoluble cellulose (crystalline cellulose) or its water-soluble forms (CMC) allows for a comprehensive assessment of cellulases or endoglucanases only, respectively. Various dyes/stains could be used to improve plate reading. They can be flooded after growth such as Congo Red and Lugol’s reagent (e.g., [193,194]), included in the medium like Congo Red (e.g., [84,195]), or used as insoluble chromogenic substrate [196]. Otherwise the β-glucosidase activity is assessed using aesculin (6,7-dihydroxycoumarin-6-O-glucoside) or arbutin (hydroquinone β-D-glucopyranoside) as substrate, leading to a chromogenic response when positive [197,198].

**Figure 11:**
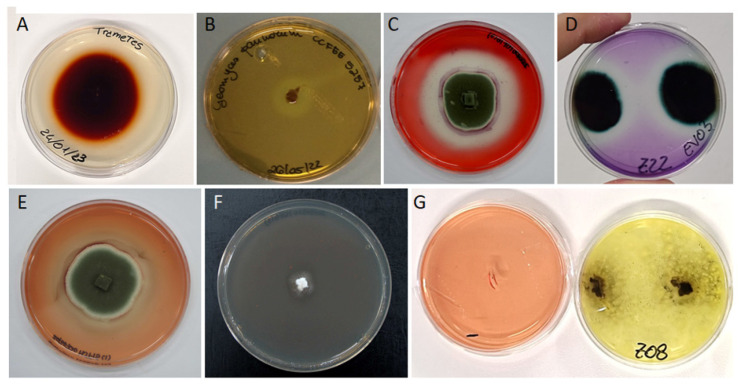
Positive responses to screening plate assays. (**A**) Laccase test (PDA guaiacol) of *Trametes versicolor* from Antarctic plant [202]; (**B**) cellulase activity (CMC agar flooded with Lugol solution) of *Pseudogymnoascus pannorum* CCFEE 5287; (**C**) cellulase activity (PDA_CMC flooded with Congo Red) of *Penicillium oxalicum*; (**D**) lignin peroxidase activity (Cz_Azure B) recorded for strain CCFFEE 10077; (**E**) xylanase activity (PDA_xylan) of *Penicillium oxalicum*; (**F**) ligninase activity (PDA lignin supplementedremazol) of *Eupenicillium rubidurum*; (**G**) Mn peroxydase activity (Cz_Phenol Red) of *Aspergillus niger* vs. negative control plate.

Xylan is generally added to common fungal media for evaluating the ability to degrade hemicellulose [83,198]. Otherwise, multiple tests can be used to assess the production of the different enzymes involved in lignin degradation. Chromogenic reactions are used to improve plate reading. For instance, in positive laccase tests, guaiacol turns red, and Remazol Brilliant Blue R changes to colorless (e.g., [75,102,199,200,201]). Similarly, the lignin peroxidase plate test employs Azure B (shifting from blue to green or colorless in positive assays, Figure 11), the Mn peroxidase test utilizes Phenol Red (turning yellow), and the phenoloxidase plate test employs gallic acid, which turns brown. These compounds are frequently added to fungal culture media such as Czapek-Dox medium (Cz) or potato dextrose agar (PDA). Table 3 reports the metabolic activities recorded for a selection of species whose presence has been documented in terrestrial WCH.

### 6.2. Instrumental Tools

To support the conservation of WCH, various instrumental tools and a range of both micro-invasive and non-invasive techniques have been developed. The preference for the latter is evidently due to the peculiarity and unicity of the work of art [2,213,214].

Microscopy offers a detailed examination of wood at various levels. Both optical and scanning electron microscopy (SEM) are frequently used to document structural wood damage and decay patterns [5,39,40,45,47,48]. Microscopy is crucial in wood identification, a fundamental step in the conservation of WCH. Identification is essential not only for understanding the original purpose of wood but also for effectively preserving WCH by distinguishing between authentic work and any subsequent repairs or alterations [8]. Macroscopic identification of woods is indeed challenging when dealing with historic artefacts [215], as many characteristics such as color, gloss, odor, weight, and structure are generally lost over time. It then becomes crucial to turn to microscopic identification. 

A definitive list of anatomical microscopic features for hardwood identification was adopted by the International Association of Wood Anatomists (IAWA) Committee in 1989 [216], and for softwood identification in 2004 [217]. These features are based both on anatomical and non-anatomical criteria. The latter include the type of growth rings, the type of vessels, their arrangement and thickenings, the presence or absence of perforation plates and their diverse morphology, the morphology of tracheids, their length, pits, and wall thickenings, as well as the presence or absence of axial parenchyma and its arrangement, and the presence of intracellular canals.

Among the non-invasive methods of analysis widely used in wooden cultural heritage, colorimetry and Fourier transform infrared (FTIR) spectroscopy play a fundamental role in the diagnosis of the conservation status and possible alteration of artworks [218,219,220,221,222].

Color measurement has been demonstrated to be a non-invasive, easy to use, and low-cost method to evaluate wood modifications. Moreover, it can be directly correlated with the chemical changes of wood components as reported in a research study by Calienno and colleagues [223]. These authors, in fact, demonstrated that the chemical alterations suffered by lignin and due to UV radiation were statistically correlated with color changes. Color is also a very important parameter for aesthetic reasons. In fact, wood species were chosen for their mechanical and durability characteristics but further for their chromatic aspect [224,225]. The method generally used for color data measurements and processing is the CIELAB where each hue is identified by three coordinates: L* that is lightness, a* and b* that are the chromatic coordinates. L* value range from 0 to 100%, a* and b* have both positive and negative values: +a* red, −a* green; +b* yellow, −b* blue. According to the international standard, color differences can be evaluate by calculating the so called ΔE* with the following formula: ΔE* = [(ΔL*)^2^ + (Δa*)^2^ + (Δb*)^2^]^½^ (EN ISO/CIE 11664-4, 2019; EN 15886, 2010) [226,227].

FTIR spectroscopy is another widely used technique in cultural heritage diagnostics for several applications ranging from material characterization to the investigation of degradation processes and the monitoring of surface modifications. Due to natural and artificial ageing under UV irradiation. FTIR is employed to investigate wood degradation due to different kinds of irradiation and biodeterioration processes [228,229,230,231,232,233]. Some authors used FTIR spectroscopy to measure the water gradient (more precisely, hydroxyl groups, OH) between the surface and the inner part of ancient and modern wooden sculptures with the aim of differentiating ancient wooden artefacts from modern ones [234]. This technique was also recently used for the characterization of biodeterioration organisms through the study of the main IR signatures due to the components of the biomass, such as polysaccharides, proteins, and lipids [181].

The main degradation of wood under UV irradiation involves lignin with chemical modification to aromatic rings. This leads to a decrease of the typical IR signature at about 1510 cm^−1^ and the consequent increase of the carbonyl band at about 1735–1740 cm^−1^ due to the formation of colored compounds responsible for the yellowing and browning of wood surfaces [223,224,229,235,236,237].

The degradation caused by biological agents may have different patterns depending on the kind of organisms, and generally occurs in both lignin and cellulose [238,239,240,241,242]. Xia and Jia demonstrated that the decay mechanisms of wood depend on the different decay organisms involved (fungi, bacteria, or insects) and the wood species. For example, *Gloeophyllum trabeum* could degrade hemicellulose selectively, while *Rhodonia placenta* could preferentially attack cellulose [242].

Some authors used FTIR spectroscopy to investigate the effect of nine decay fungi on beech wood for possible application in biotechnology [243]. These authors demonstrated the different rates of cellulose and lignin degradation by the nine chosen fungi using the ratio of the main infrared signal of lignin (1504 cm^−1^) and those of carbohydrates at cm^−1^: 1732, 1367, 1155, and 895 [243].

The presence of fungi in artworks has also been correlated with the detection of calcium oxalate (Ca-oxalate) crystals that were attributed to the action of oxalic acid by the fungi on the artwork constituent materials [244]. Ca-oxalate is frequent on artworks’ deteriorated surfaces. Particularly hard to remove with different kind of systems, its detection is relevant in cultural heritage restoration. In the above-mentioned paper, FTIR analysis showed that the depolymerization of cellulose takes place in the early stages of wood decay, suggesting an early-stage colonization by brown rot fungi. The authors further demonstrated that a biomineralization process occurs after colonization by fungi. In this process, as long as the organic components of the wood are destroyed, inorganic salts appear and gradually transform into other salts. They concluded that the knowledge of these decay patterns could supply relevant information to choose the appropriate conservation treatments for artworks [244].

The use of FTIR spectroscopy was also addressed in the evaluation of the age of wooden artefacts [245]. In this paper the authors present a method for wood dating analyzing the chemical breakdown of wood components. They propose a prediction model covering a maximum of 3000 years, including old living trees and construction wood.

Raman spectroscopy has also been used as molecular spectroscopy to study wood degradation [230,246], even if this technique is not commonly used to investigate wooden materials [213]. 

Lastly, pulse compression thermography (PuCT) should be cited as an innovative technique recently applied to investigate wood panel paintings to reveal inner degradation patterns [247,248]. PuCT is a non-invasive imaging technique that is able to perform the inspection of artworks from the surface to inner parts. In the case of panel paintings, the complete thickness of the support can be investigated. In a recent paper, this technique proved useful to detect and map wood support degradation due to insect galleries [249]. In respect to other techniques used for the inspection of wooden supports, PuCT has the advantage of producing images at different instant times corresponding to different depths of the wooden support. Moreover, being inherently an imaging procedure, PuCT can easily scan large surfaces; it does not require safety protocols, as in the case of X-ray based techniques, and, with the recent increase in camera performance, a very high 2-D resolution and frame rate can be reached [249]. 

## 7. Present Issues and Future Perspectives in WCH Conservation

### 7.1. Fungal Diversity—Evidence Looking Ahead

Wood constitutes a significant portion of the world’s historical and artistic heritage. Multidisciplinary expertise is essential for studying the deterioration and preservation of wooden artefacts involving fields such as engineering, architecture, chemistry, physics, and biology. As a consequence of this multidisciplinarity, only a small fraction of the papers produced in the last 25 years provide taxonomical information on fungi affecting WCH. Moreover, these data cannot be considered exhaustive, as searches in scientific repositories could be hindered by language barriers. Indeed, as highlighted in a previous study [250], some peer-reviewed papers cannot be retrieved as they are published in different languages and alphabets. 

The taxonomical changes applied over time and the different identification methods introduce a margin of uncertainty around species identifications. Anyhow, fungal diversity recorded on terrestrial WCH raises two main findings. One pertains to the present knowledge of this item. The other concerns the prevalence and distribution of some taxa.

Notably, about 23% of the recorded taxa were identified only at the genus, family, or order level, indicating our limited knowledge of fungal diversity, the challenges in gathering new data, and potential methodological biases. Beyond the expected new taxa from extreme environments (polar regions were part of the study), others could be related to technical issues in acquiring knowledge. Investigations of wood-decaying basidiomycetes have highlighted difficulties in identifying them from cultures, obtaining pure cultures, and then reliable reference sequences [176,177,178]. Ascomycota generally do not pose similar culture-based problems, but the challenge of comparing them with reliable sequences persists. Samson and colleagues evidenced how the abundant information produced in recent decades on fungal taxonomy, physiology, and ecology, fragmented across numerous articles and books, has led to the publication and reuse of outdated and/or incorrect data [251]. Despite heightened constraints aimed at improving the quality of deposited sequences, a portion of the data stored in databases lacks total reliability. An incorrectly identified sequence could lead to further errors or interfere as background noise, making it challenging to find a reliable closest match. What enables us to discriminate quickly between more and less reliable deposited data is the voucher number/collection number, which uniquely defines the considered strain in terms of its origin and traceability. Collections of microorganisms, especially if accredited to the World Federation for Culture Collections (WFCC), can be helpful, if not basic in giving solid bases for research [178,252,253,254]. On the other hand, identification could intentionally be left unexplored because it is considered to be outside the research scope or due to technical bias. A reduced accuracy of molecular identification could be tied, indeed, to the target region considered, sequence length, and the reference strain used for comparison [147,255]. In fact, it is known that sequencing the actin gene (*actA*) and translation elongation factor EF-1α (*TEF1*) for the *Cladosporium* genus, β-tubulin (*BT2*) and calmodulin (*cmdA*) genes for *Aspergillus* and *Penicillium* can yield better results in identification, even though ITS has been recognized as the primary barcode for fungi [256,257,258,259]. Correct identification is difficult but is at the base of all information [251]. In this, like in other contexts related to the conservation of cultural heritage, enhanced resolution in organism identification could allow for a better assessment of both degradation potential and associated risks [260]. 

About the fungal occurrence, records on Mucoromycota were limited. Notably, Basidiomycota prevail on wooden architectural heritage (WAH) coming frequently from continental (Dfb, Dfc) and temperate oceanic (Cfb) climate regions. Various wood rotting species are shared among these geoclimatic areas, such as, for example, *Antrodia sinuosa*, *Serpula lacrymans*, *Gloeophyllum abietinum*, *Gloeophyllum trabeum*, *Stereum hirsutum*, and *Trametes versicolor*, to name a few. Others, such as *Bjerkandera adusta* and *Dacrymyces stillatus*, are found also in harsh areas (e.g., Chile [121]). Meanwhile, *Coniophora puteana* has been found even in Antarctica, demonstrating broad adaptability skills and extended risk potential. All the listed species are quite common even in common houses, with a dominance of *Serpula lacrymans* in Europe and the United States [261,262,263]. Nevertheless, the verified threshold parameters that would be useful to prevent their growth is limited to just a few species.

Ascomycota can thrive in dry environments and are commonly observed in situations where brown and white rot are hindered, leading to soft rot decay in harsh environments [204]. From decayed huts in Antarctica, the most frequently isolated species belong to the *Cadophora* genus (e.g., *Cadophora fastigiata*, *Cadophora malorum*) [38,45,72,86,123,124]. At the global scale, the most frequent Ascomycota recorded on WCH belong mainly to *Aspergillus*, *Penicillium*, and *Cladosporium* genera represented by 38, 52, and 14 identified species, respectively. While *Alternaria*, *Trichoderma* and *Talaromyces* accounted 17, 12, and nine identified species, respectively. Their competitiveness and sporulating rate favor their broad distribution worldwide. *Cladosporium cladosporioides* was recorded even from polar regions (BCDE) evidencing its cold loving trait, while *Aspergillus flavus*, *Aspergillus niger*, *Aspergillus ochraceus*, and *Aspergillus versicolor* (ABCD) were frequent even in tropical environments. Conversely, the distribution of *Alternaria alternata*, *Aspergillus fumigatus*, and *Aspergillus terreus* (BCD) respect to *Trichoderma viride* (ACD) could reflect higher moisture requirements from the latter. A separate mention is necessary for *Chaetomium* spp. given their long-acknowledged involvement in soft rot decay [36,264]. In detail, *Chetomium globusus* has been recorded with wider distribution than *C. elatum*, being repeatedly isolated from hot desertic climate areas [110], in addition to temperate and continental climatic areas.

Taxa distribution along with metabolic features could offer insights into the potential detrimental risks associated with different species, even if some traits exhibit considerable variability within the same species or genus [265]. Unfortunately, the harmful potential of the majority of species isolated from lignocellulosic artefacts remains largely unknown, despite the significance of this information for the conservation of various heritage artefacts, including books and textiles and new materials [266,267,268]. This knowledge can be readily obtained through reliable, rapid, simple, and cost-effective metabolic plate tests. Consequently, the necessity to invest in collections of microorganisms becomes even more critical and relevant. Indeed, collections facilitate the direct integration of knowledge concerning vouchered/reference strains, aligning directly and promptly with scientific and technological progress. In a rapidly advancing world where big data are collected and managed for in silico scientific purposes, there is still a place for traditional microbiology, and culture collections represent a resource not only for biotechnological applications but also for laying the foundations for a better interpretation of big data. 

### 7.2. Fungal Succession in Wood Decay and Wood-Staining Fungi

Fungal succession plays a crucial role in the wood biodeterioration process and is necessary for the development of wood-rotting fungi [93]. Wood degradation should be considered as the result of metabolism, species co-occurrence, and interactions within the settling community. Due to the complexity of these events, this process is largely unknown, and the evolution of degradative processes is unpredictable [269]. Nevertheless, the role of opportunist species has been suggested since most of the wood-degrading fungi that digest cellulose and lignin require a long period of wet conditions to successfully colonize wooden substrata [270]. The basic process has been described on natural wood in which decomposition is promoted by latent fungal inhabitants of living wood becoming primary colonizers just after tree death. These opportunists, utilizing available sugars, hemicellulose, proteins and amino acids could increase substrate bioreceptivity, creating favorable conditions for wood-decaying fungi [92]. The airborne spores competitively replace primary colonizers, while late-stage wood decomposition is characterized by cord-forming fungi [271]. In field tests, the duration of rainfall was assumed to be a more crucial factor than the rainfall sum in the decay progression, along with constant temperature [153]. Nevertheless, each succession event is unique as it depends on the material features and its environment. Prevention is better than cure. To pose a foundation for preventive measures, it is necessary to categorize and then broaden the understanding of heritage artefacts, encompassing aspects such as wood type (often overlooked in the reviewed papers), treatments employed to improve durability, pictorial layer preparation, pigments used, and more. Then, instrumental diagnostic tools can be used for preventive strategies anticipating potential damage instead of limiting their role to post-damage assessment. For the characterization of the surrounding environment, much has been done in monitoring physical measures (e.g., humidity and temperature), as well as in identifying potential agents of degradation in areas relevant to the asset to be preserved (e.g., [138]). Since air circulation could favor contamination, indoor and outdoor aerobiological studies have been performed [70,272,273,274]. Nevertheless, more efforts are needed to integrate these data for identifying general and specific shared guidelines, allowing for the comparison of results and learning from shared experiences. 

For WCH, even non-structural alterations such as discolorations must be avoided due to the high staining potential of melanins. The involvement of *Cladosporium* species, *Aureobasidium pullulans* (including *A. melanogenum* [275]), and *Sydowia polyspora* in wood color alterations is well-established [23], but they were not the only black fungi (BF) recorded on WCH.

In the field of cultural heritage conservation, BF have been frequently associated to stone materials [276,277] even if they were found also in subterranean environments and wall paintings [194,278,279]. The large-scale data collection conducted in this study evidenced the recurrent presence of BF, even from WCH. 

*Exophiala xenobiotica*, previously isolated from hydrocarbon-contaminated sites using both long–cold incubation and enrichment protocols [280,281,282], has also been isolated multiple times from wood samples collected from polar regions [45,72,85,86]. This evidence, along with its confirmed cellulase activity [192,275], suggests a possible link with the soil-saprobic trait even at low temperatures. The finding of chaethothyrialean taxa (e.g., *Capronia*, *Cladophialophora* sp., *Exophiala* sp., *Rhinocladiella* spp.) was not limited to polar regions anyway [90,94]. The isolation of *Knufia* is no surprise since this genus has been recorded in Antarctica from soil and stone [283,284], and the closest tested species showed cellulase activity [207]. Occasionally, the presence of other dothideomycetous BF has been recorded. This includes strains belonging to the Neodevriesiaceae family [71] and *Constantinomyces* genus [127]. *Zalaria obscura* [137] and *Pseudotaeniolina globosa* [90,107] were also recorded. The latter was also isolated from experiments on early wood colonization [285], raising further interest in BF.

The interest in BF lies in their ability to tolerate several physical and chemical stresses, including biocidal treatments [286,287]. Species belonging to the genera *Aureobasidium*, *Cladosporium*, *Exophiala*, and *Rhinocladiella* were also found to cope with highly toxic wood preservatives such as creosote, a complex mixture of chemicals including polycyclic aromatic hydrocarbons (PAHs) and arsenic, and CCA (chromated copper arsenate) [280,288,289,290,291]. This is not surprising since these BF were repeatedly found in hydrocarbon-contaminated areas and revealed tolerance to heavy metals [292]. Due to environmental issues these preservatives have been replaced by alternative copper-based compounds such as alkaline copper quat (ACQ) and copperazole (CA), quats such as didecyldimethylammonium chloride (DDAC), and the isothiazolone 4,5-dichloro-2-n-octyl-4-isothiazol-3-one (DCOIT), and IPBC (3-iodo-2-propynylbutylcarbamate) [293]. The tolerance of BF against copper-based biocides and quats has already been reported [207,294], but data on specific products for wood treatments are still scant and deserve further investigation.

### 7.3. WCH Conservation and World Climate Change

Floods, extreme rainfall, permafrost thawing, rising sea levels, storm surges, extreme heat, and droughts are only a few examples of the ongoing changes in the natural environment tied to climate change [295]. The rise in temperatures in polar environments creates more favorable conditions for growth [296]. This can lead to an imbalance in the resident microflora towards more competitive species that accelerate the wood degradation process, also extending their period of activity [73]. Elevated relative humidity in a warmer climate accelerates the biological decay of cultural heritage, particularly affecting historical wooden buildings. Prolonged wet periods combined with rising temperatures create favorable conditions for various biological activities such as biomass accumulation, fungal decay, and insect infestations with particular concern about termites [297]. The potential consequences include structural collapse in the case of timber buildings and the spread of termite attacks toward northern regions, covering a wider geographical range [297,298,299]. Extreme events, such as heavy rainfall and pluvial flooding, are expected to increase across Europe, particularly in urban areas [300]. Conversely, in Italy, these events are likely to alternate with extreme droughts [301]. These rapid and drastic shrinking and swelling changes lead to significant physical stress on wood. They accelerate weathering processes, making structures less solid and more prone to biological attacks because of increased bioreceptivity [302]. 

Controlling temperature and humidity levels is crucial in indoor environments, particularly in climatic regions where mild temperatures and high humidity encourage fungal growth. However, this preventive practice is not always applicable because museums and artworks are often housed in historic and religious buildings [156]. Sudden increases in RH can occur regardless of the strict monitoring protocols in place. Both new and historic structures can be affected, and water damage (e.g., roof and gutter flaws, and rising damp) or malfunctions in the climate control system can occur (e.g., HVAC, dehumidifiers, fan coils, etc.) [113,156,303]. The impact of climate change will significantly affect the indoor climate of aged, drafty buildings lacking HVAC systems [304], resulting in heightened energy consumption elsewhere. Energy costs should not be overlooked because of the current energy crisis started in 2022. The decrease in indoor temperatures by 1 or 2 degrees during the cold season, as suggested in numerous European countries, has led to colder internal surfaces, leading to an increased risk of condensation and fungal stroke [151].

Other preventative measures are aimed at reducing external contamination. As acknowledged, fungal propagules can enter an interior environment not only through entrance doors but also via visitors and staff themselves, along with their shoes and clothing, serving as primary pathways [92,305]. In addition, incorrectly operating air filtration systems (normally used to reduce microbial load) and air conditioning systems may also serve as sources of fungal propagules [92,156,306]. Dust, containing both microbial contaminants and nutrients, should be regularly removed for preventive purposes. However, this practice is often underestimated [307]. In this scenario, the present spores could act opportunistically in the event of sudden environmental changes. Xerophilic and xerotolerant organisms, with a low water requirement threshold, might germinate rapidly, initiating surface colonization and preparing the substrate to succession. Thus, *Aspergillus*, even though it is recognized as a significant threat to cultural heritage conservation [308], should not be the only one. Indeed, by extension, so are all fungi with extreme traits such as living under drought conditions, the ability to cope with wood preservatives, growing at low temperatures, even below zero, or withstanding long periods of dormancy [133,274,307]. So that not only those fungi capable of rapid exploits deserve attention, but it also necessary to deepen our knowledge on those able to grow with little (oligotrophs) for long periods unnoticed. All these organisms represent a significant threat, even in formally safe storerooms (RH < 60%, aw < 0.6) when local reduced ventilation and/or wrapping lead to the creation of pockets of moisture conducive to their development [120,274].

More efforts are needed to improve the predictive power of aerobiological outdoor and indoor investigations coupled with complementary surfaces’ microbiological data and microclimatic reliefs. Even from stroke analysis it is possible to understand their dynamics, and to enhance buffering systems during critical conditions. This is essential for designing short-, medium-, and long-term strategies with the intention of curbing to contrast the imminent deterioration of collections utilizing effective and low-energy-cost engineering solutions. This is because, during extreme climatic events, electricity could be limited in availability or not available at all [113]. 

Different studies have provided evidence that fungi that are dangerous for modern materials and constructions are in large part the same as those that jeopardize the integrity of ancient artefacts [66,138,139,148]. Therefore, it is necessary to consider these two closely interconnected areas and life sciences as indispensable gears for the preservation of all materials because no matter how much effort is made to increase the performance and durability of materials, microorganisms and especially fungi will always find a way to feed on them.

## 8. Conclusions

The biodeterioration of materials represents the crossroads between the past (represented by WCH) and the present, with modern construction/manufacturing practices oriented towards ecological sustainability and/or energy efficiency. For this reason, the interconnection between these fields should be improved. Life scientists could bridge knowledge gaps on fungal diversity, ecological traits, and involvement in decay dynamics, providing solid foundations for studying material deterioration and conserving cultural heritage. In this regard, collections of microorganisms and databases that can accommodate all relevant biological data (e.g., genes, genome) play a crucial role, and deserve attention and support. In light of the occurring climatic changes, more preventive shared protocols and warning limits are needed.

## Figures and Tables

**Table 1:** List of the fungal taxa retrieved from the reviewed papers. Strain names are reported according to Index Fungorum (last checked on 20 December 2023). Information on the type of artefact, location, and corresponding references is provided. Accession numbers of ITS, LSU (bold), and SSU (bold blue) sequences are reported. An alternate background color has been used to distinguish the different phyla represented here in the following order: Ascomycota (grey), Basidiomycota (white), and Mucoromycota (grey).

Fungal Species	Wooden Artefact	Location	References	Accession Nr
*Acremonium murorum* (Corda) W. Gams	concentration camp barracks	Auschwitz, POL	[67,69]	
*Acremonium sclerotigenum* (Moreau & R. Moreau ex Valenta) W. Gams	concentration camp barracks	Auschwitz, POL	[69]	
*Acremonium* sp.	concentration camp barracks, woodblocks, hunting lodge	Auschwitz, POL; Haeinsa, KOR; Saint Germain-en-Laye, FRA	[67,70,71]	MG920347, MG920349
*Acrodontium antarcticum* Cabello	historic structures	Whalers Bay, ATA	[72]	KC514872
*Alpinaria* sp.	archaeological remains	West Greenland, DNK	[73]	
*Alternaria alternata* (Fr.) Keissl	concentration camp barracks, various artworks, Madonna, frame, Virgin Mary, sculptures, church, indoor church surface, funerary equipment, church, mosque	Auschwitz, POL; Montefeltro, ITA; Bratislava, SVK; Šid, SRB; Gora, HRV; Naples, ITA; Bucharest, ROM; Bârzava, ROM; Saqqara, EGY; Amărăşti, ROM; Cairo, EGY	[67,74,75,76,77,78,79,80,81,82]	
*Alternaria angustiovoidea* E.G. Simmons	stored woodblocks, pipe organ, historic structures; timber structures; woodblocks	Hapcheon, KOR; Spišká Nová Ves, SVK; Cape Evans, Cape Royds, Allan Hill, Lake Fryxell, ATA; Snow Hill, ATA; Uiwang, Gonju, and Suncheon, KOR	[83,84,85,86,87]	KJ002062, EU520086, JF439434, KF558883; JF802106; DQ317386; JN084021; JF439435, JN084022; JF439435
*Alternaria aspera* Woudenb. & Crous	expedition hut	Fort Conger, CAN	[47]	MW033332
*Alternaria astragalicola* W. Zhao, Q. Ning & J.Y. Yan	church walls	Nicula, ROM	[88]	KC415805
*Alternaria atra* (Preuss) Woudenb. & Crous	expedition hut	Fort Conger, CAN	[47]	MW033333
*Alternaria botrytis* (Preuss) Woudenb. & Crous	concentration camp barracks	Auschwitz, POL	[67,69]	
*Alternaria chartarum* Preuss	frames, icons and walls	Šid, SRB; Nicula, ROM	[76,88]	KC292360
*Alternaria conjuncta* E.G. Simmons	church	Calen, CHI	[89]	KF638549
*Alternaria malorum* (Ruehle) U. Braun, Crous & Dugan	expedition hut	Fort Conger, CAN	[45]	MW033334
*Alternaria multiformis* (E.G. Simmons) Woudenb. & Crous	historic structures	Whalers Bay ATA	[72]	KC514886
*Alternaria oudemansii* (E.G. Simmons) Woudenb. & Crous	historic structures	Snow Hill, ATA	[86]	FJ235987
*Alternaria papavericola* Woudenb. & Crous	stair salt mine	Hallstatt, AUT	[90]	KR081404
*Alternaria pogostemonis* M. Luo, M.P. Zhao & Z.Y. Dong	printing woodblocks	Hapcheon, KOR; Hadong, KOR	[83,91]	DQ491088; JQ907485
*Alternaria radicina* Meier, Drechsler & E.D. Eddy	concentration camp barracks	Auschwitz, POL	[69]	
*Alternaria rosae* E.G. Simmons & C.F. Hill	church	Nercon, CHI	[89]	KF638556
*Alternaria* sp.	woodblocks, sculptures, funerary boats, locomotive turntable, frames, pirogue, wooden face pipe organs, historic building; historic building	Haeinsa, KOR; Belgrade, SRB; Giza and Saqqara, EGY; La Plata, ARG; Šid, SRB; Tianjin, CHN; Cairo, EGY; Bad Ish and Vienna, AUT; Havana, CUB; Huşi, ROM	[70,76,80,92,93,94,95,96,97,98]	
*Alternaria tellustris* (E.G. Simmons) Woudenb. & Crous	expedition hut	Fort Conger, CAN	[45]	MW033335
*Alternaria tenuissima* (Kunze) Wiltshire	statues	Bratislava, SVK	[99]	
*Antarctomyces psychrotrophicus* Stchigel & Guarro	historic structures	Whalers Bay, ATA	[72]	KC514843
*Apiospora arundinis* (Corda) Pintos & P. Alvarado	printing wood blocks	Hapcheon, KOR; Ulsan, KOR	[83,100]	KJ361492; HQ380772
*Apiospora aurea* (Calvo & Guarro) Pintos & P. Alvarado	woodblocks	Seosan, KOR	[101]	**AB195633**
*Apiospora guangdongensis* C.F. Liao & Doilom	woodblocks	Gonju, KOR; Hadong, KOR	[87,91]	HQ647335; HQ832803
*Apiospora qinlingensis* (C.M. Tian & N. Jiang) Pintos & P. Alvarado	historic structures	Chilean Station, ATA	[72]	KC514845
*Apiospora rasikravindrae* (Shiv M. Singh, L.S. Yadav, P.N. Singh, Rah. Sharma & S.K. Singh) Pintos & P. Alvarado	woodblocks	Ulsan, KOR	[100]	JN198505
*Apiospora sacchari* (Speg.) Pintos & P. Alvarado	printing woodblocks, statues	Hapcheon, KOR; Bratislava, SVK	[93,99]	HQ115646
*Apiospora sphaerosperma* (Pers.) Pintos & P. Alvarad	polychromatic sculpture	Lomnička, SVK	[102]	
*Arthrinium* sp.	woodblocks	Haensia, KOR	[70]	
*Arthrobotrys superba* Corda	historic structures	Chilean Station, ATA	[72]	KC514844
*Ascocoryne albida* (Berk.) Seifert	historic structures	Whalers Bay, ATA	[72]	KC514846
*Aspergillus* sp.	artworks, frame, parts of the Solar boat, pirogue, pipes organ, historic building, historic building, woodblocks, altar sculpture, church iconostasis, puppet; church, organ pipes and sculpture, woodblocks	Montefeltro, ITA; Cairo, EGY; Giza, EGY; Tianjin, CHN; Schwanenstadt, AUT; Havana, CUB; Boianu Mare, ROM; Ulsan, KOR; Lomnička, SVK; Spălanca, ROM; Palermo, ITA; Huşi, ROM; Reichenbach and Zwickau, GER; Yeongju, KOR	[74,80,94,96,97,98,100,102,103,104,105,106,107]	HQ443246; GU797139
*Aspergillus amstelodami* (L. Mangin) Thom & Church	Madonna, coffin cover, mask, wooden statues, painted ceiling	Bratislava, SVK; Saqqara, EGY; Cairo, EGY; Bratislava, SVK; Zillis, CHE	[75,80,99,108]	
*Aspergillus brasiliensis* Varga, Frisvad & Samson	Frame and coffin; coffin cover; funerary boat	Cairo, EGY; Saqqara, EGY; Saqqara, and Giza, EGY	[80]	
*Aspergillus candidus* Link	artworks	Montefeltro, ITA	[74]	
*Aspergillus chevalieri* Thom & Church	mask, pipe organ	Cairo EGY; Spišská Nová Ves, SVK	[80,84]	KX400781
*Aspergillus clavatus* Desm.	concentration camp barracks, wood blocks, woodblocks	Auschwitz, POL; Yeongju, KOR; Seoul, KOR	[67,101,107]	AB008398, EU273880; **M55626**
*Aspergillus cristatus* Raper & Fennel	pipe organ	Spišská Nová Ves, SVK	[84]	JQ743649
*Aspergillus egyptiacus* Moub. & Moustafa	coffin cover	Saqqara, EGY	[80]	
*Aspergillus fischeri* Wehmer	altar panel	Bratislava, SVK	[75]	
*Aspergillus flavipes* (Bainier & R. Sartory) Thom & Church	frame, woodblocks	Cairo, EGY; Sokcho, KOR	[80,101]	**FJ458446**
*Aspergillus flavus* Link	concentration camp barracks, indoor barracks, Madonna, stored sculptures, clapper; part of Solar boat, frame and coffin cover; mosque, statues; box, frames; wall cover, sculpture, coffins and statues fragments, historic building, woodblock, painted chair	Auschwitz, POL; Auschwitz, POL; Bratislava, SVK; Belgrade, SRB; Troaş-Săvârşin, ROM; Giza, Cairo, and Saqqara, EGY; Cairo, EGY; Bratislava, SVK; Wando, KOR; Šid, SRB; Huşi, ROM; Palermo, ITA; Abydos; EGY; Havana, Cuba; Hapcheon, KOR; Havana, Cuba;	[67,69,75,76,79,80,82,91,92,96,109,110,111,112,113]	MK095969, MK095970, MK095971, MK095977; OQ820160; **KF562195**
*Aspergillus fumigatus* Fresen.	concentration camp barracks, indoor barracks, frame; crucifixion and frame; coffins and statue basement	Auschwitz, POL; Auschwitz, POL; Šid, SRB; Naples, ITA; Abydos, EGY	[67,69,76,78,110]	**MK095978, MK095979**
*Aspergillus glabripes* F. Sklenář, Jurjević & Hubka	pipe organ	Waldzell, AUT	[96]	MH424912
*Aspergillus glaucus* (L.) Link	concentration camp barracks, indoor barracks, artworks, frame, mosque	Auschwitz, POL; Auschwitz, POL; Montefeltro, ITA; Cairo, EGY; Cairo, EGY	[67,69,74,80,82]	
*Aspergillus janus* Raper & Thom	sculptures and painted chair	Havana, CUB	[113]	
*Aspergillus jensenii* Jurjević, S.W. Peterson & B.W. Horn	woodblocks	Hapcheon, KOR	[100]	KC339215
*Aspergillus megasporus* C.M. Visagie, N. Yilmaz & K.A. Seifert	icons and church walls; historic structures	Nicula, ROM; Cape Evans and Cape Royds, ATA	[85,89]	KC009789, DQ317335
*Aspergillus melleus* Yukawa	indoor barracks	Auschwitz, POL	[69]	
*Aspergillus minisclerotigenes* Vaamonde, Frisvad & Samson	mosque	Cairo, EGY	[114]	KU243046
*Aspergillus nidulans* (Eidam) G. Winter	artworks, concentration camp barracks, coffin cover	Montefeltro, ITA; Auschwitz, POL; Saqqara, EGY	[67,74,80]	
*Aspergillus niger* (Eidam) G. Winter	concentration camp barracks, artworks, Madonna, frames, church, Solar boat parts, church, mosque, woodblocks, statues, wooden face, stored statues, woodblocks; coffins and statues; wall cover, painted chair, mosque; stored desk, cashbox, sarcophagus, museum walls, artefacts	Auschwitz, POL; Montefeltro, ITA; Bratislava, SVK; Šid, SRB; Lunca Moţilor, ROM; Giza, EGY; Amărăşti, ROM; Cairo EGY; Gonju, KOR; Belgrade, SRB; Cairo, EGY; Bratislava, SVK; Seosan, KOR; Abydos, EGY; Havana, CUB; Havana, CUB; Cairo, EGY; Tianjin, CHN; Wando, KOR; Cairo, EGY; Makasar, IDN; Irbid, JOR;	[67,74,75,76,79,80,81,82,87,92,95,99,101,110,111,113,114,115,116,117,118,119]	KU243044; (KM979775, AM270052, AM269986); **MK095975**, **MK095989**; OQ820164; HQ589136; **EU667998**
*Aspergillus ochraceus* K. Wilhelm	coffin, woodblocks, stairs salt mine, stored sculptures, statue and coffins; frame	Cairo, EGY; Uiwang, KOR Hallstatt; AUT; Belgrade, SRB; Abydos, EGY; Havana, CUB	[80,87,90,92,110,111]	KR081402; KF435031
*Aspergillus oerlinghausenensis* Bader & Houbraken	stored woodblocks	Hapcheon, KOR	[83]	FJ867935
*Aspergillus parasiticus* Speare	coffin and Solar boat parts	Cairo and Giza, EGY	[80]	
*Aspergillus penicillioides* Spegazzini	artworks, pipe organ	Montefeltro, ITA; Eggelsberg and Altheim, AUT	[74,96]	MH424903, MH424909, MH4249408
*Aspergillus pseudoglaucus* Blochwitz	stair salt mine	Hallstatt, AUT	[90]	KR081398
*Aspergillus repens* (Corda) Sacc.	sculptures	Belgrade, SRB	[92]	
*Aspergillus restrictus* G. Smith	stairs salt mine	Hallstatt, AUT	[90]	KR081407
*Aspergillus ruber* (Jos. König, Spieck. & W. Bremer) Thom & Church	icons and walls; woodblocks	Nicula, ROM; Sokcho, KOR	[88,101]	**KC009779**; **AY004346**
*Aspergillus salinarum* (Greiner, Peroh, Weig & Rambold) Zalar & Greiner	stair salt mine	Hallstatt, AUT	[90]	KR081418, KR081419, KR081422
*Aspergillus salisburgensis* Zalar, Martinelli & Piñar	stair salt mine	Hallstatt, AUT	[90]	KR081397, KR081420, KR081421
*Aspergillus sclerotiorum* G.A. Huber	woodblocks, stored sculpture	Ulsan, KOR; Coimbra, PRT	[100,120]	AY373866
*Aspergillus siamensis* Manoch, Eamvijarn & Yaguchi	woodblocks	Ulsan, KOR	[100]	AB674770
*Aspergillus sibiricus* V.A. Iliushin	woodblocks	Yeongju, KOR	[107]	KJ746594
*Aspergillus sydowii* (Bainier & Sartory) Thom & Church	pipe organ	Spišská Nová Ves, SVK	[84]	KT151589
*Aspergillus terreus* Thom	altar panel and Madonna, church; coffin, Solar boat parts and mask; mosque, statues, mask, woodblocks	Bratislava, SVK; Bucharest, ROM; Saqqara, Giza, and Cairo, EGY; Cairo, EGY; Bratislava, SVK; Abydos, EGY; Hapcheon, KOR	[75,79,80,82,99,110,112]	**MK095983**, **MK095976**; OQ820163; **KC762934**
*Aspergillus tubingensis* Mosseray	mosque	Cairo, EGY	[114]	KU243047
*Aspergillus ustus* (Bainier) Thom & Church	statues, altar sculpture, sculptures	Bratislava, SVK; Bušovce, SVK; Havana, CUB	[99,102,113]	
*Aspergillus versicolor* (Vuill.) Tirab.	concentration camp barracks; artworks, sculpture and frame; pipe organ, mask, coffin lid and funerary boat; wooden sculptures; stairs salt mine, woodblocks; woodblocks, woodblocks; sculpture and painted chair, stored statues	Auschwitz, POL; Montefeltro, ITA; Naples, ITA; Spišká Nová Ves, SVK; Cairo and Saqqara, EGY; Hallstatt, AUT; Hapcheon, KOR; Andong, KOR; Seoul, KOR; Havana, Cuba; Coimbra, PRT	[67,74,78,80,84,90,100,101,107,113,120]	KR081417; KX082930; JN545818; AB008411; **AF548067**
*Aureobasidium insectorum* Q.M. Wang, F. Wu & M.M. Wang	locomotive turntable, saltpeter works	La Plata, ARG; Humbertone and Santa Laura, CHI	[92,121]	JF817344
*Aureobasidium pullulans* (de Bary & Löwenthal) G. Arnaud	concentration camp barracks, indoor barracks, woodblocks, external hunting cabins	Auschwitz, POL; Auschwitz, POL; Haeinsa, KOR; Spitsbergen, SJM	[67,69,70,122]	
*Bahusandhika terrestris* (P.C. Misra) J.L. Crane, S. Hughes & A.N. Mill.	concentration camp barracks	Auschwitz, POL	[69]	
*Beauveria bassiana* (Bals.-Criv.) Vuill.	Madonna, statues	Bratislava, SVK; Bratislava, SVK	[75,99]	
*Blastobotrys arbuscula* de Hoog, Rant.-Leht. & M.T. Sm.	hunting lodge	Saint Germaine-en-Laye, FRA	[71]	MH411229
*Botryosphaeria* sp.	concentration camp barracks	Auschwitz, POL;	[67,69]	
*Botryotrichum domesticum* D.W. Li & N.P. Schultes	stables near expedition hut wall	Cape Royds, ATA	[123]	GU212411, GU212412, GU212414-GU212420
*Botrytis cinerea* Pers.	concentration camp barracks, woodblocks	Auschwitz, POL; Haeinsa, KOR	[69,70]	
*Botrytis* sp.	church iconostasis	Boianu Mare, ROM	[103]	
*Cadophora* aff. *Gregata* (Allington & D.W. Chamb.) T.C. Harr. & McNew	roof board of stables and boxes	Cape Royds, ATA	[123]	GU212431, GU212433, GU212434
*Cadophora* aff. *luteo-olivacea*	expedition huts, expedition hut wall-stables,	Cape Evans ATA; Cape Royds, ATA	[38,123]	GU212388, GU212390; AY371506
*Cadophora* aff. *malorum* I	expedition hut wall, stables, and boxes	Cape Royds, ATA	[123]	GU212379, GU212380
*Cadophora* aff. *malorum* II	boxes and stables under roof boards	Cape Royds, ATA	[123]	GI212384-GU212387
*Cadophora fastigiata* Lagerb. & Melin	timber structure, expedition huts; historic structures; external wall, stables	Mc Graw hut, New Harbor-ATA; Fort Conger, CAN; Whalers Bay, ATA; View Point, Horseshoe Island, ATA; Cape Royds, ATA	[38,45,72,86,123]	GU212369-GU212373; MW033336; AY371511; KF514850, KF589024; FJ235940, FJ235980
*Cadophora indistincta* Q.M. Wang, B.Q. Zhang & M.M. Wang	expedition hut	Fort Conger, CAN	[45]	MW033368
*Cadophora luteo-olivacea* (J.F.H. Beyma) T.C. Harr. & McNew	expedition hut; expedition hut; historic structures; historic structures; wooden structures; hut—gallery wall	Cape Evans, ATA; Fort Conger, CAN; Whalers Bay, ATA; Cape Royds, New Harbor, Mount Fleming, Dry Walleys and Lake Fryxell, ATA; Port Lockroy, Detaille Island, ATA; Cape Evans, ATA	[38,45,72,85,86,124]	MW033337; AY371507, AY371508, AY371509, AY371510; KF514851 DQ317327; FJ235941
*Cadophora malorum* (Kidd & Beaumont) W. Gams	stables and boxes, structure remains, expedition hut, historic structures, timber structures, lower bunk wall	Cape Royds, ATA; Fort Conger, CAN; Cape Royds and Cape Evans, ATA; Cape Evans, Cape Royds, New Harbor, Mount Fleming - Ross Sea, ATA; View Point, Port Lockroy, Wordie House, Detaille Island, Horseshoe Island, East Base, Base E, ATA Cape Evans, ATA	[38,45,85,86,123,124]	GU212375, GU212377, GU212378; MW033338; AY371503, AY371504 AY371505; DQ317328; FJ235942
*Cadophora rotunda* L. Mostert, R. van der Merwe, Halleen & Gramaje	historic structures	Cape Evans, Cape Royds -Ross Sea, ATA	[85]	DQ317326
*Cadophora* sp.	expedition hut; archeological remains; hut-gallery wall	Fort Conger, CAN; West Greenland DNK; Cape Evans, ATA	[45,73,124]	MW033375
*Candida digboiensis* G.S. Prasad, Mayilraj, Sood & Ban. Lal	church	Rome, ITA	[125]	
*Candida zeylanoides* (Castell.) Langeron & Guerra	timber structures	East Base, ATA	[86]	FJ235945
*Capronia* cft *pulcherrima* (Munk) E. Müll., Petrini, P.J. Fisher, Samuels & Rossman	expedition hut	Fort Conger, CAN	[45]	MW033339
*Capturomyces* aff. *funiculosus* S. Bien, C. Kraus & Damm	expedition huts; timber structures	New Harbor ATA; Horseshoe Island, ATA	[38,86]	AY371513; FJ235938
*Chaetomidium* sp.	archaeological remains; historical building	West Greenland, DNK; Havana, CUB	[73,97]	
*Chaetomium elatum* Kunze	concentration camp barracks; hunting lodge; wooden statues	Auschwitz, POL; Saint Germain-en-Laye, FRA Bratislava, SVK	[67,71,99]	
*Chaetomium globosum* Kunze	concentration camp barracks, indoor barracks; Madonna; door, beams, statues; box, stored desk, statues, stored sculptures; statue, polymateric artwork	Auschwitz, POL; Auschwitz, POL; Bratislava, SVK; Belgrade, SRB; Bratislava, SVK; Abydos, EGY; Tianjin, CHN; Coimbra, PRT; La Plata, ARG; Tuscania, ITA	[67,69,75,92,99,110,115,120,126,127]	KX379227, KP671732, KC485058; OQ231609
*Chaetomium* sp.	artworks, Solar boat parts, pirogue, polychromatic sculpture	Montefeltro, ITA; Giza, EGY; Tianjin, CHN; Lomnička, SVK	[74,80,94,102]	
*Chaetomium subglobosum* Sergeeva	walls	Nicula, ROM	[88]	JN209930
*Chrysosporium* sp.	archeological remains, stables	West Greenland, DNK Cape Royds, ATA	[73,123]	GU212405, GU212406
*Cladophialophora* aff. *humicola*	historic structures	Whalers Bay, ATA	[72]	KC514855
*Cladorrhinum hyalocarpum* (Arx) X. Wei Wang & Houbraken	doors old houses	Fez, MAR	[128]	
*Cladosporium anthropophilum* Sand.-Den., Gené & Wiederh.	artefact fragments	Abydos, EGY	[110]	MK095987
*Cladosporium benschiae* P.P. Costa, A.W.C. Rosado & O.L. Pereira	pirogue	Tianjin, CHN	[94]	HQ148094
*Cladosporium cladosporioides* (Fresen.) G.A. de Vries	expedition hut, concentration camp barracks, indoor barracks, artworks, Madonna and polychromatic statue, frames, sculpture and frame, funerary boat, church, statues, altar sculpture, fragments, boxes, gallery wall, old doors	Fort Conger, CAN; Auschwitz, POL; Auschwitz, POL; Montefeltro, ITA; Bratislava, SVK; Šid, SRB; Naples, ITA; Saqqara, EGY; Amărăşti, ROM; Bratislava, SVK; Bušovce, SVK; Abydos, EGY; Cape Royds, ATA; Cape Evans, ATA; Fez, MAR	[45,67,69,74,75,76,78,80,81,99,102,110,123,124,128]	GU212394; MW033340; **MK095984, MK095986**
*Cladosporium herbarum* (Pers.) Link	concentration camp barracks; indoor barracks, sarcophagus	Auschwitz, POL; Auschwitz, POL; Giza, EGY	[67,69,117]	
*Cladosporium hillianum* Bensch, Crous & U. Braun	historic structures, timber structures, woodblocks	Cape Evans, Cape Royds, New Harbor, Allan Hill, ATA; Snow Hill, Horseshoe Island, East Base, ATA; Ulsan, KOR	[85,86,100]	DQ317332; FJ235947; AJ300331
*Cladosporium limoniforme* Bensch, Crous & U. Braun	artefact fragments	Abydos, EGY	[110]	MK095991
*Cladosporium ossifragi* (Rostr.) U. Braun & K. Schub.	timber structures	Snow Hill, ATA	[86]	FJ235946, FJ235951
*Cladosporium oxysporum* Berk. & M.A. Curtis	concentration camp barracks; sculpture and frame	Auschwitz, POL; Naples, ITA	[69,78]	
*Cladosporium perangustum* Bensch, Crous & U. Braun	pipe organ	Spišká Nová Ves, SVK	[84]	FJ490621
*Cladosporium pulvericola* Bensch & Samson	stair salt mine	Hallstatt, AUT	[90]	KR081401
*Cladosporium* sp.	concentration camp barracks, hunting lodge, archaeological remains, artworks, frame, altar, altar; frame, coffin cover, and Solar boat parts; locomotive turntable, pirogue, pipe organ, historic building, church iconostasis, polymateric artwork gilded woodcarving	Auschwitz, POL; Saint Germain-en-Laye, FRA; West Greenland, DNK; Montefeltro, ITA; Šid; SRB; Bucharest, ROM; Bârzava, ROM; Cairo, Saqqara and Giza, EGY; La Plata, ARG; Tianjin, CHN; Schwenenstadt, AUT; Havana, CUB; Boianu Mare, ROM; Tuscania, ITA; Evora, PRT	[67,71,73,74,76,79,80,93,94,96,97,103,127,129]	
*Cladosporium sphaerospermum* Penzig	concentration camp barracks, stair salt mine	Auschwitz, POL Hallstatt, AUT	[67,90]	KR081410
*Cladosporium vicinum* Bensch & Samson	timber walls	Nicula, ROM	[88]	KC865299
*Cladosporium xylophilum* Bensch, Shabunin, Crous & U. Braun	structures saltpeter works	Humbertone and Santa Laura, CHI	[121]	JF499838
*Cladosporium tenuissimum* Cooke	frames	Šid, SRB	[76]	
*Clavicipitaceae* sp.	historic structures, church	Whalers Bay, ATA; Castro, CHI	[72,89]	KF638547; KC514858
*Coniochaeta acaciae* M.C. Samar., Gafforov & K.D. Hyde	expedition hut	Fort Conger, CAN	[45]	MW033341, MW033344
*Coniochaeta africana* Damm & Crous	woodblocks	Haensa, KOR; Yeongju, KOR	[70,107]	JX910082
*Coniochaeta cipronana* Coronado-Ruiz, Avendaño, Escudero-Leyva, Conejo-Barboza, P. Chaverri & Chavarría	church walls	Nicula, ROM	[88]	
*Coniochaeta deborreae* Hern.-Restr.	expedition hut, timber structures	Fort Conger, CAN; View Point, ATA	[45,86]	MW033342; FJ2359990
*Coniochaeta hoffmannii* (J.F.H. Beyma) Z.U. Khan, Gené & Guarro	expedition hut, historic structures, timber structures	Fort Conger, CAN; Whalers Bay and Chilean Station, ATA; Wordie House, Horseshoe Island, East Base, ATA	[45,72,86]	MW033345; KC514856; FJ235948, FJ235955
*Coniochaeta marina* Dayar., S. Tibell, Tibell & K.D. Hyde	historic structures	Chilean Station, ATA	[72]	KC154861
*Coniochaeta nivea* A.E. Arnold, A.H. Harr., Y.K. Huang, J.M. U’Ren, Massimo, Knight-Connoni & Inderb	historic structures	Whalers Bay and Chilean Station, ATA	[72]	KC514871
*Coniochaeta* sp.	expedition hut, historic structures, stables	Fort Conger, CAN; Cape Evans, ATA; Cape Royds, ATA	[45,85,123]	GU212367, GU212368; MW033343; DQ317353
Coniochaetaceae sp.	historic structures	Whalers Bay, ATA	[72]	KC514860
*Coniothecium* sp.	historic timber structures	Kizhi, RUS	[130]	
*Coniothyrium ferrarisianum* Biga, Cif. & Bestagno	historic structures	Whalers Bay—Deception Island, ATA	[72]	KC154883, KC514884
*Coniothyrium telephii* (Allesch.) Verkley & Gruyter	expedition hut; historic structures	Fort Conger, CAN; Whalers Bay, ATA	[45,72]	MW033346; KC514880
*Constantinomyces* sp.	polymateric artwork	Tuscania, ITA	[127]	OQ231607
*Cordycipitaceae* sp.	hunting lodge	Saint Germaine-en-Laye, FRA	[71]	MG920352
*Cosmospora* sp.	archaeological remains; timber structures	West Greenland, DNK; Wordie House, ATA	[73,86]	FJ235966
*Cosmospora viridescens* (C. Booth) Grafenhan & Seifert	expedition hut, historic structures, timber structures	Fort Conger, CAN; Cape Evans, Cape Royds, ATA; Hope Bay, View Point, Snow Hill, Port Lockroy, Wordie House, Detaille Island, Horseshoe Island, East Base, ATA	[45,85,86]	MW033347; DQ317333; FJ235967
*Curvularia lunata* (Wakker) Boedijn	concentration camp barracks	Auschwitz, POL	[69]	
*Curvularia* sp.	historic building	Havana, Cuba	[97]	
*Cytospora brevispora* (G.C. Adams & Jol. Roux) G.C. Adams & Rossman	church	Achao, CHI	[89]	KF638546
*Daldinia childiae* J.D. Rogers & Y.M. Ju	woodblocks	Seosan, KOR	[101]	GQ906966
*Dasyscyphus* sp.	open-air museum	Riga, LAV	[66]	
*Debaryomyces hansenii* (Zopf) Lodder & Kreger-van Rij	timber structures	Snow Hill, Port Lockroy, Wordie House, ATA	[86]	FJ235952
*Dialonectria ullevolea* Seifert & Gräfenhan	historic structures	Cape Royds, ATA	[85]	DQ317342
*Diatrypella longiasca* L.S. Dissan., J.C. Kang & K.D. Hyde	woodblocks	Gonju, KOR	[87]	
*Dichotomopilus dolichotrichus* (L.M. Ames) X. Wei Wang & Samson	historic structures	Cape Royds, ATA	[85]	DQ317331
*Didymella glomerata* (Corda) Qian Chen & L. Cai	concentration camp barracks, historic structures	Auschwitz, POL; Mount Fleming, Lake Frywell, New Harbor, Alla Hill, ATA	[69,85]	DQ317367
*Dissoconiaceae* sp.	woodblocks	Andong, KOR	[107]	KC986373
*Drechslera* sp.	frames	Šid, SRB	[76]	
*Entimomentora* sp.	historic structures	Whalers Bay and Chilean Station, ATA; Hope Bay and East Base, ATA	[72,86]	KC514887; FJ235965
*Epicoccum nigrum* Link	concentration camp barracks, indoor barracks, frames, frame, printing woodblocks, pipe organ	Auschwitz, POL; Auschwitz, POL; Šid, SRB; Cairo, EGY; Hapcheo, KOR; Spišká Nová Ves, SVK	[67,69,76,80,83,84]	KF573983, EU272494
*Epicoccum* sp.	woodblocks, woodblocks, locomotive turntable	Hapcheo, KOR; Gonju, KOR; La Plata, ARG	[83,87,93]	FJ788133, FJ788133; KC215136
*Eupenidiella venezuelensis* (Crous & U. Braun) Quaedvl. & Crous	structures saltpeter works	Humbertone and Santa Laura, CHI	[121]	HQ115663
*Eurotiales* sp.	hunting lodge	Saint Germaine-en-Laye, FRA	[71]	MG920353
*Eurotium* sp.	historic building	Havana, CUB	[97]	
*Exophiala* sp.	salt mine stair, pirogue	Hallstatt, AUT; Tianjin, CHN	[90,94]	KR081415
*Exophiala xenobiotica* de Hoog, J.S. Zeng, Harrak & Deanna A. Sutton	expedition hut; historic structures; historic structures; timber structures	Fort Conger, CAN; Whalers Bay, ATA; Cape Royds, ATA; View Point, Detaille Island, Horseshoe Island, ATA	[45,72,85,86]	MW033348; KC514859; DQ317336; FJ235954
*Fusarium* sp.	concentration camp barracks, indoor barracks, woodblocks, pipes organ, historic building	Auschwitz, POL; Auschwitz, POL; Haeinsa, KOR; Schwanenstadt, AUT; Havana, Cuba	[67,69,70,96,97]	
*Fusarium annulatum* Bugnicour	stored woodblocks	Hapcheo, KOR	[83]	GU066624
*Fusarium avenaceum* (Fr.) Sacc.	sarcophagus	Giza, EGY	[117]	
*Fusarium* cft. *solani* (Mart.) Sacc.	museum stored desk	Tianjin, CHN	[115]	KT876643, FJ345352, LT615323
*Fusarium foetens* Schroers, O’Donnell, Baayen & Hooftman	historic structures	Cape Evans andCape Royds, ATA	[85]	DQ317368
*Fusarium oxysporum* Schltdl.	Madonna, wood box lid	Naples, ITA; Abydos, EGY	[78,110]	MK095974
*Fusarium poae* (Peck) Wollenw.	sarcophagus	Giza, EGY	[117]	
*Fusarium reticulatum* Mont.	stored woodblocks	Hapcheo, KOR	[83]	JX402186
*Gamszarea kalimantanensis* (Kurihara & Sukarno) Z.F. Zhang & L. Cai	structures saltpeter works	Humbertone and Santa Laura, CHI	[121]	KC311469
*Graphium rubrum* Rumbold	historic structures	Whalers Bay and Chilean Station, ATA	[72]	KF514852
*Graphium silanum* Goidànich	expedition hut	Fort Conger, CAN	[45]	
*Gregarithecium curvisporum* Kaz. Tanaka & K. Hiray.	woodblocks	Yeongju, KOR	[107]	AB807547
*Hamatocanthoscyphaceae* sp.	historic structures	Whalers Bay, ATA	[72]	KC514866
*Harzia* sp.	sculptures	Havana, Cuba	[113]	
Helotiales sp.	historic structures; archaeological remains; historic structures	Chileanan Station -, ATA; West Greenland, DNK; Cape Evans, ATA	[72,73,85]	KC514865; DQ317329
*Humicola fuscoatra* Traaen	Indoor barracks	Auschwitz, POL	[67,69]	
*Humicola* sp.	hunting lodge	Saint Germaine-en-Laye, FRA	[71]	
*Hyaloscypha leuconica* (Cooke) Nannf.	sacred buildings	Latgale region, LAV	[66]	
*Hyaloscyphaceae* sp.	historic structures	Whalers Bay and Chilean Station, ATA	[72]	KC514869, KC514888, KC514890
*Hypocreales* sp.	stair salt mine	Hallstatt, AUT	[90]	KR081399, KR081413, KR081414
*Hypoxylaceae* sp.	woodblocks	Hapcheon, KOR	[100]	FJ481153
*Hypoxylon* sp.	woodblocks	Yangseon, KOR	[91]	GU166476
*Isaria* sp.	archaeological remains	West Greenland, DNK	[73]	
*Jeremyomyces labinae* Crous & R.K. Schumach.	historic structures	Whalers Bay, ATA	[72]	KC514876
*Juxitiphoma eupyrena* (Sacc.) Valenz.-Lopez, Crous, Stchigel, Guarro & J.F. Cano	expedition hut	Fort Conger, CAN	[45]	MW033350
*Knufia* sp.	expedition hut	Fort Conger, CAN	[45]	MW033376
*Lachnum* sp.	expedition hut, historic structures	Fort Conger, CAN; Whalers Bay, ATA	[45,72]	MW033351; KC514889
*Lecanicillium praecognitum* Gorczak & Kisło	church	Rilan, CHI	[89]	KF675189
*Lecanicillium* sp.	woodblocks	Haeinsa, KOR; Ulsan, KOR	[70,100]	KF766521
*Leptodontidium beauverioides* (de Hoog) de Hoog	expedition huts; historic structures	Discovery hut, ATA; Cape Royds, ATA	[38,85]	AY371512; DQ317330
*Leuconeurospora capsici* (J.F.H. Beyma) Malloch, Sigler & Hambl.	historic structures	Discovery hut, ATA	[85]	DQ317347
*Leuconeurospora* sp.	timber structures	Hope Bay, View Point, Horseshoe Island, East Base, Base E, ATA;	[86]	FJ235937
*Metapochonia bulbillosa* (W. Gams & Malla) Kepler, S.A. Rehner & Humber	church	Calen, San Juan, CHI	[89]	KF638550, KF638558
*Microascus trigonosporus* C.W. Emmons & B.O. Dodge	woodblocks	Seoul, KOR	[101]	KT587319
*Microdochium nivale* (Fr.) Samuels & I.C. Hallett	mosque	Cairo, EGY	[82]	
*Mollisia* aff. *gibbospora* Kušan, Matočec, Pošta, Tkalčec & Mešić	historic structures	Whalers Bay, ATA	[72]	KF514847
*Mollisia* aff. *undulatodepressula* (Feltgen) Le Gal & F. Mangenot	expedition hut	Fort Conger, CAN	[45]	MW033352
*Mollisia ligni* (Desm.) P. Karst.	historic structures	Whalers Bay, ATA	[72]	KF514874
*Mollisia* sp.	historic structures	Whalers Bay, Chilean Station, ATA	[72]	KF514848, KF514849, KF514873
*Mollisiaceae* sp.	historic structures	Cape Evans, Cape Royds, ATA	[85]	DQ317334
*Myriodontium keratinophilum* Samson & Polon	concentration camp barracks	Auschwitz, POL	[69]	
*Nectriaceae* sp.	historic structures	Whalers Bay, ATA	[72]	KC514857
*Neodevriesiaceae* sp.	hunter lodge	Saint Germaine-en-Laye, FRA	[71]	MG920346
*Neostagonaspora* sp.	historic structures	Whalers Bay, ATA	[72]	KC154877
*Neurospora crassa* Shear & B.O. Dodge	stored sculptures	Belgrade, SRB	[92]	
*Neurospora sitophila* Shear & B.O. Dodge	artworks	Montefeltro, ITA	[74]	
*Neurospora* sp.	historic building	Havana, CUB	[97]	
*Niesslia* sp.	archaeological remains	West Greenland, DNK	[73]	
*Nigrospora oryzae* (Berk. & Broome) Petch	concentration camp barracks; statue	Auschwitz, POL; La Plata, ARG	[67,126]	
*Nigrospora* sp.	historic building	Havana, Cuba	[97]	
*Ochrocladosporium frigidarii* Crous & U. Braun	expedition hut	Fort Conger, CAN	[45]	MW033357
*Oidiodendron griseum* Robak	expedition hut; church	Fort Conger, CAN; Rome, ITA	[45,125]	MW033358
*Oidiodendron* sp.	historic structures, timber structures	Whalers Bay, ATA; East Base, ATA	[72,86]	KC514875; FJ235968
Onygenales sp.	woodblocks	Andong, KOR	[107]	FJ545752
*Orbicula parietina* (Schrad.) S. Hughes	timber structures	Wordie House, ATA	[86]	FJ235991
*Orbilia auricolor* (A. Bloxam) Sacc.	timber structures	Base E, ATA	[86]	FJ235988
*Paecilomyces maximus* C. Ram	pipe organ	Spišká Nová Ves, SVK	[84]	KJ207406
*Paecilomyces* sp.	coffin cover, historic building, historic building	Cairo, EGY; Havana, Cuba; Huşi, ROM	[80,97,98]	
*Paecilomyces variotii* Bainier	concentration camp barracks; sculpture	Auschwitz, POL; Gora, HRV	[69,77]	
*Paramicrothyrium chinense* H.X. Wu & K.D. Hyde	woodblocks	Yeongju, KOR	[107]	JQ036224
*Paraphaeosphaeria* sp.	stored woodblocks	Hapcheon, KOR	[83]	JQ936270
*Paraphoma fimeti* (Brunaud) Gruyter, Aveskamp & Verkley	historic structures, timber structures	Whalers Bay, ATA; Hope Bay, Snow Hill, Port Lockroy, Wordie House, Horseshoe Island, ATA	[72,86]	KC514881; FJ235989
*Parengyodontium album* (Limber) C.C. Tsang, J.F.W. Chan, W.M. Pong, J.H.K. Chen, A.H.Y. Ngan, Cheung, C.K.C. Lai, D.N.C. Tsang, S.K.P. Lau, P.C.Y. Woo	concentration camp barracks, indoor barracks, hunting lodge, salt mine stair, woodblocks, sculptures	Auschwitz, POL; Auschwitz, POL; Saint Germain-en-Laye, FRA; Hallstatt, AUT; Ulsan, KOR; Havana, Cuba	[67,69,71,90,100,113]	KR081409; MH411228, MG920350; JF779670
*Patinella* sp.	archaeological remains	West Greenland, DNK	[73]	
*Penicillium* aff. *violaceum* (Sopp) Sacc	polymateric artwork	Tuscania, ITA	[127]	OQ231607
*Penicillium albocoremium* (Frisvad) Frisvad	walls; timber structures	Nicula, ROM; East Base and Base E, ATA	[86,88]	KC009832; FJ235977
*Penicillium auratiogriseum* Dierckx	statues	Bratislava, SVK	[99]	
*Penicillium bialowiezense* K.W. Zaleski	hunting lodge	Saint Germaine-en-Laye, FRA	[71]	MG923832
*Penicillium brevicompactum* Dierckx	pipe organ, polymateric artwork	Vienna, AUT; Tuscania, ITA	[96,127]	OQ231610
*Penicillium brocae* S.W. Peterson, Jeann. Pérez, F.E. Vega & Infante	woodblocks	Hadong, KOR	[91]	DQ123642
*Penicillium canescens* Sopp.	expedition hut	Fort Conger, CAN	[45]	MW033359
*Penicillium capsulatum* Raper & Fennell	coffin cover	Cairo, EGY	[80]	
*Penicillium carolineherscheliae* Y.P. Tan, Bishop-Hurley & R.G. Shivas	woodblocks	Suncheon, KOR	[87]	JX296565
*Penicillium cavernicola* Frisvad & Samson	timber structures	Hope Bay, Snow Hill, Wordie House, Detaille Island, East Base, Base E, ATA	[86]	FJ235975
*Penicillium cerradense* Cruvinel, Magalhães, P. O. Pinho	structures saltpeter works, gilded woodcarving;	Humbertone and Santa Laura, CHI, Aveiro, PRT	[121,131]	JQ734919, AY232277
*Penicillium chermesinum* Biourge	mosque	Cairo, EGY	[82]	
*Penicillium chrysogenum* Thom	artworks, altar panel, Madonna, Virgin Mary sculpture, polychrome crucifixion and frame, church pronaos, altar, stair salt mine, pipe organ, statues, polychromatic sculptures, Mosque, objects doors old house	Montefeltro, ITA; Bratislava, SVK; Bratislava, SVK; Gora, HRV; Naples, ITA; Bârzava, ROM; Troaş-Săvârşin, ROM; Hallstatt, AUT; Eggelsberg, AUT; Bratislava, SVK; Lomnička, SVK; Cairo, EGY; Irbid, Jordan; Fez, MAR	[74,75,77,78,79,90,96,99,102,114,119,128]	KR081405, KU243045
*Penicillium citreonigrum* Dierckx	concentration camp barracks; concentration camp barracks; hunting lodge; wooden icons and walls; temple surfaces	Auschwitz, POL; Auschwitz, POL; Saint Germain-en-Laye, FRA; Nicula, ROM; Haeinsa, KOR	[67,69,71,88,132]	JN689966
*Penicillium citreosulfuratum* Biourge	printing woodblocks	Suncheon, KOR; Ulsan, KOR; Hapcheon, KOR	[87,100,112]	GU934551; EU497959, EU497942
*Penicillium citrinun* Thom.	concentration camp barracks, indoor barracks, artworks, woodblocks, sarcophagus, stored sculpture	Auschwitz, POL; Auschwitz, POL; Montefeltro, ITA; Ulsan, and Hapcheon, KOR; Giza, EGY; Coimbra, PRT	[67,69,74,100,117,120]	KF530869; KF758800, JX192960
*Penicillium commune* Thom	concentration camp barracks, indoor barracks; Madonna; church, pipes organ, statues, woodblocks, doors old house	Auschwitz, POL; Auschwitz, POL; Bratislava, SVK; Castro, CHI; Vienna, AUT; Bratislava, SVK; Seoul, KOR; Fez, MAR	[67,69,75,89,96,99,101,127]	KF638548; KX580630
*Penicillium copticola* Houbraken, Frisvad & Samson	stored sculpture	Coimbra, PRT	[120]	
*Penicillium corylophilum* Dierckx	expedition hut, indoor barracks, indoor church surfaces; woodblocks	Fort Conger, CAN; Auschwitz, POL; Troaş-Săvârşin, Juliţa, and Lunca Moţilor, ROM; Hapcheon, KOR	[45,69,79,100]	MW033360; AY373906
*Penicillium crustosum* Thom.	pipe organ; woodblocks, doors old house	Spišká Nová Ves, SVK; Suncheon, KOR; Fez, MAR	[84,87,128]	HQ262521; JQ011376
*Penicillium decumbens* Thom	woodblocks	Yangsan, KOR	[91]	KP132491
*Penicillium dierckxii* Biourge	indoor barracks	Auschwitz, POL	[69]	
*Penicillium digitatum* (Pers.) Sacc.	church, structures saltpeter works	Bucharest, ROM; Humbertone and Santa Laura, CHI	[79,121]	AB479307
*Penicillium echinulatum* Biourge	hut-wall near floor	Cape Evans, ATA	[124]	
*Penicillium expansum* Link	church surface, church, pipes organ, statues, hut- near floor, doors old house	Juliţa, ROM; Calen, CHI; Vienna, AUT; Bratislava, SVK; Cape Evans, ATA; Fez, MAR;	[79,89,96,99,124,128]	KF638552
*Penicillium fimorum* Frisvad & Houbraken	expedition hut	Fort Conger, CAN	[45]	MW033361
*Penicillium flavigenum* Frisvad & Samson	expedition hut	Fort Conger, CAN	[45]	MW033362
*Penicillium fundyense* Visagie, David Clark & Seifert	hunting lodge	Saint Germaine-en-Laye, FRA	[71]	MG920354
*Penicillium fuscoglaucum* Biourge	timber structures	View Point, Snow Hill, ATA	[86]	FJ235971
*Penicillium glandicola* (Oudem.) Seifert & Samson	woodblocks	Ulsan, KOR	[100]	NR_119395
*Penicillium granulatum* Bainier	woodblocks, doors old house;	Gonju, KOR; Fez, MAR	[87,128]	AY373916
*Penicillium griseofulvum* Dierckx	salt mine stair	Hallstatt; AUT	[90]	KR081408
*Penicillium herquei* Bainier & Sartory	Madonna and statues	Bratislava, SVK	[75,99]	
*Penicillium limosum* S. Ueda	woodblocks	Sokcho, KOR	[101]	EF413620
*Penicillium melinii* Thom	concentration camp barracks	Auschwitz, POL	[67]	
*Penicillium multicolor* Grig. Man. & Porad	woodblocks	Hadong, KOR	[91]	JN799647
*Penicillium oregonense* Visagie & Samson	hunting lodge	Saint Germaine-en-Laye, FRA	[71]	MG920333
*Penicillium oxalicum* Currie &Thom	woodblocks, stored desk, stored sculpture	Hapcheo, KOR; Tianjin, CHN; Coimbra, PRT	[83,115,120]	KC344971; KP868627, KF152942, FJ358409
*Penicillium pimiteouiense* S.W. Peterson	temple surfaces	Haeinsa, KOR	[132]	
*Penicillium roqueforti* Thom	Madonna, timber structures	Bratislava, SVK; Horseshoe Island, East Base, ATA	[75,86]	AB027410; FJ235974
*Penicillium rubens* Biourge	Structures saltpeter works, barrel remain	Humbertone and Santa Laura, CHI; Livingstone Island, ATA	[121,133]	JQ015265; MZ318091
*Penicillium rubidurum* Udagawa & Y. Horie	pirogue	Tianjin, CHN	[94]	MK551157
*Penicillium sacculum* E. Dale	statues	Kremnica and Bratislava, SVK	[99]	
*Penicillium senticosum* D.B. Scot	stored woodblocks	Hapcheo, KOR	[83]	HQ607978
*Penicillium* sp.	concentration camp barracks, woodblocks, hunting lodge, archaeological remains, artworks, frames, Naos, coffins and funerary objects, church surface, pirogue, pipe organ, historic building, church iconostasis, woodblocks, woodblocks, altar sculpture and Virgin Mary, church; church; historic building; woodblocks, coffins and statue basement, gilded woodcarving, stored sculpture, Wawel villa beams	Auschwitz, POL; Haeinsa, KOR; Saint Germain-en-Laye, FRA; West Greenland, DNK; Montefeltro, ITA; Šid, SRB; Bârzava, ROM; Cairo and Saqqara, Giza, EGY; Spălanca, ROM; Tianjin, CHN; Schwanenstadt, AUT; Havana, CUB; Boianu Mare, ROM; Ulsan, KOR; Yeongju, KOR; Lomnička and Bušovce, SVK; Amărăşti, and Huşi, ROM; Seosan, KOR; Abydos, EGY; Coimbra, PRT; Evora, PRT; Rabka-Zdrój, POL	[67,70,71,73,74,76,79,80,81,94,96,97,98,100,101,102,103,105,110,120,129,134]	HM469401, JN157800; **MF040753**, **HM469401**
*Penicillium* aff. *spathulatum* Frisvad & Samson	structures saltpeter works	Humbertone and Santa Laura, CHI	[121]	JF439503
*Penicillium speluncae* Visagie & N. Yilmaz	historic structures; timber structures	Cape Royds, Discovery hut, New Harbor, ATA; Snow Hill, Wordie House, Detaille Island, East Base, ATA	[85,86]	DQ317344; FJ235973
*Penicillium stoloniferum* Thom	expedition hut, timber structures	Fort Conger, CAN; View Point, ATA	[45,86]	MW033365; FJ235969
*Penicillium sumatraense* Svilv.	woodblocks, pirogue,	Gonju, KOR; Tianjin, CHN	[87,94]	KT310939; HE962603
*Penicillium swiecickii* K.W. Zaleski	expedition hut	Fort Conger, CAN	[45]	MW033366
*Penicillium verrucosum* Dierckx	stored sculptures	Belgrade, SRB	[92]	
*Penicillium glabrum* (Wehmer) Westling	expedition hut, church walls, woodblocks	Fort Conger, CAN; Nicula, ROM; Hadong, KOR	[45,88,91]	MW033363; KC797645; JQ863239
*Penicillium samsonianum* L. Wang, Frisvad, Hyang B. Lee & Houbraken	expedition hut	Fort Conger, CAN	[45]	MW033364
*Periconia byssoides* Pers	stored woodblocks	Hapcheo, KOR	[83]	KC954160
*Pestalotiopsis* sp.	woodblocks	Haeinsa, KOR	[70]	
*Peziza cerea* Sowerby	balcony beam	Latgale region, LAV	[66]	
*Peziza domiciliana* Cooke	historical house	Targu Neamt, ROM	[135]	
*Phacidiales* sp.	historic structures	Whalers Bay, Chilean Station, ATA	[72]	KC514853
*Phialemonium atrogriseum* (Panas.) Dania García, Perdomo, Gené, Cano & Guarro	expedition hut, historic structures; historic structures; timber structures	Fort Conger, CAN; Whalers Bay, ATA; Cape Royds, ATA; Port Lockroy, ATA	[45,72,85,86]	MW033367; KC514842; DQ317343; FJ235936
*Phialemonium* sp.	archaeological remains; woodblocks	West Greenland, DNK; Ulsan, KOR	[73,100]	HE610370
*Phialocephala dimorphospora* W.B. Kendr.	historic structures	Whalers Bay, Chilean Station, ATA	[72]	KC514878
*Phialocephala lagerbergii* (Melin & Nannf.) Grünig & T.N. Sieber	historic structures	Whalers Bay, ATA	[72]	KC514879
*Phialocephala mallochii* Tanney & B. Douglas	timber structures	Wordie House, ATA	[86]	FJ235978
*Phialophora hyalina* (W. Gams) Unter. & Réblová	expedition hut	Fort Conger, CAN	[45]	MW033369
*Phialophora* sp.	archaeological remains	West Greenland, DNK	[73]	
*Phoma hebarum* Westendorp	expedition hut	Fort Conger, CAN	[45]	MW033370
*Phoma* sp.	Solar boat parts; timber structures	Giza, EGY; View Point, Snow Hill, East Base, ATA	[80,86]	FJ235979
*Pithomyces chartarum* (Berk & M.A. Curtis)	concentration camp barracks, stored wooden sculpture from museum	Auschwitz, POL; Coimbra, PRT	[67,120]	
*Pleosporaceae* sp.	historic structures, church walls, church	Whalers Bay, ATA; Nicula, ROM; San Juan, CHI	[72,88,89]	KF638538; EU479954; KC514891
*Pleosporales* sp.	woodblocks	Hadong, KOR	[91]	HQ696060
*Pochonia* sp.	expedition hut; timber structures	Fort Conger, CAN; Wordie House, ATA	[45,86]	MW033371; FJ235949, FJ235963
*Polyphilus sieberi* Ashrafi, D.G. Knapp, W. Maier & Kovács	expedition hut	Fort Conger, CAN	[45]	MW033372
*Preussia* sp.	historic structures	Cape Evans, ATA	[85]	DQ317341
*Pseuderotium* sp.	archaeological remains	West Greenland, DNK	[73]	
Pseudeurotiaceae sp.	historic structures	Whalers Bay, ATA	[72]	KC514882
*Pseudogymnoascus* sp.	historic structures, archaeological remains, wall at the lower bunk	Whalers Bay and Chilean Station, ATA; Western Greenland, DNK; Cape Evans, ATA	[72,73,124]	KC514864
*Pseudogymnoascus appendiculatus* A.V. Rice & Currah	timber structures	Hope Bay, Horseshoe Island, East Base, ATA	[86]	FJ235957
*Pseudogymnoascus pannorum* (Link) Minnis & D.L. Lindner	expedition hut, Madonna, stables, historic structures; statues, timber structures	Fort Conger, CAN; Bratislava, SVK; Cape Royds, ATA; Cape Evans, Cape Royds, Dry Valleys and Lake Fryxell, ATA; Bratislava, SVK; Port Lockroy, Detaille Island, East Base, ATA	[45,75,85,86,99,123]	GU212402; MW033373; DQ317337, DQ317339; FJ235959, FJ235960
*Pseudogymnoascus verrucosus* A.V. Rice & Currah	stables - roof board stables; timber structures	Cape Royds, ATA; Snow Hill, Port Lockroy, Horseshoe Island, East Base and Base E, ATA;	[86,123]	GU212396, GU212397, GU212423- GU212425, GU212428; FJ235958
*Pseudotaeniolina globosa* De Leo, Urzì & de Hoog	salt mine stairs, woodblocks	Hallstatt, AUT; Yeongju, KOR	[90,107]	KR081416; GU214520
*Purpureocillium lilacinum* (Thom) Luangsa-ard, Houbraken, Hywel-Jones & Samson	expedition hut, indoor barracks, gilded carved wood	Fort Conger, CAN; Auschwitz, POL; Aveiro, PRT	[45,69,131]	MW033374; JQ734918
*Pyrenophora biseptata* (Sacc. & Roum.) Crous	artworks	Montefeltro, ITA	[74]	
*Ramichloridium* sp.	pirogue	Tianjin, CHN	[94]	
*Rhinocladiella atrovirens* Nannf.	timber structures	Wordie House, ATA	[86]	FJ235983
*Rhinocladiella similis* de Hoog & Calig.	timber structures	View Point, ATA	[86]	FJ235935
*Sarea difformis* (Fr.) Fr.	historic structures	Discovery Hut, ATA	[85]	DQ317349
*Sarocladium kiliense* (Grütz) Summerb	timber structures	Snow Hill, ATA	[86]	FJ235956
*Sarocladium strictum* (W. Gams) Summerb.	concentration camp barracks	Auschwitz, POL	[67,69]	
*Scleroconidioma* sp.	woodblocks	Haeinsa, KOR	[70]	
*Scleroconidioma sphagnicola* Tsuneda, Currah & Thormann	church	Quinchao, CHI	[89]	KF638553
*Scopulariopsis sphaerospora* Zach	mask	Cairo, EGY	[80]	
*Scytalidium lignicola* Pesante	church	Montalcino, ITA	[125]	
*Scytalidium* sp.	churches	Calen, San Juan, and Quinchao, CHI	[89]	KF638551, KF638554; KF638555; KF638557
*Sordaria fimicola* (Roberge ex Desm.) Ces. & De Not.	concentration camp barracks indoor barracks, Virgin Mary sculpture	Auschwitz, POL; Auschwitz, POL; Gora, HRV	[67,69,77]	
*Stachybotrys chartarum* (Ehrenb.) S. Hughes	salt mine stairs	Hallstatt, AUT	[90]	KR081400
*Stemphylium botryosum* Wallr.	funerary boat	Saqqara, EGY	[80]	
*Stemphylium paludiscirpi* E.G. Simmons	artefact fragment	Abydos, EGY	[110]	MK095988
*Stemphylium vesicarium* (Wallr.) E.G. Simmons	artefact fragment	Abydos, EGY	[110]	MK095985, MK095990
*Stysanus* sp.	timber structures	Kizhi, RUS	[130]	
*Surculiseries* sp.	woodblocks	Seoul, KOR	[101]	AB014045
*Sydowia polyspora* (Bref. & Tavel) E. Müll	expedition hut; woodblocks; historical structures; historic structures; timber structures; hut - wall above frost	Fort Conger, CAN; Haeinsa, KOR; Whalers Bay, ATA; Cape Evans, Discovery Hut, ATA; View Point, Horseshoe Island, East Base, Base E, ATA; Cape Evans, ATA	[45,70,72,85,86,124]	MW033377; KC514868; DQ317340; FJ235953, FJ235964, FJ235984
*Talaromyces* sp.	pirogue	Tianjin, CHN	[94]	
*Talaromyces aerugineus* (Samson) N. Yilmaz, Frisvad & Samson	woodblocks	Seoul, KOR	[101]	DQ365947
*Talaromyces domesticus* Jurjević & S.W. Peterson	whaling boat remains	Livingstone Island, ATA	[133]	MZ318092, MZ223864
*Talaromyces duclauxii* (Delacr.) Samson, N. Yilmaz, Frisvad & Seifert	coffin cover	Cairo, EGY	[80]	
*Talaromyces flavus* (Klöcker) Stolk & Samson	pirogue	Tianjin, CHN	[94]	KU216713
*Talaromyces fusiformis* A.J. Chen, Frisvad & Samson	stored woodblocks	Hapcheo, KOR	[83]	JQ988819
*Talaromyces liani* (Kamyschko) N. Yilmaz, Frisvad & Samson	woodblocks, woodbox	Hapcheon, KOR; Abydos, EGY	[100,110]	MK095973; JX677940
*Talaromyces pinophilus* (Hedgc.) Samson, N. Yilmaz, Frisvad & Seifert	sculpture fragments	Abydos, EGY	[110]	MK095980
*Talaromyces rugulosus* (Thom) Samson, N. Yilmaz, Frisvad & Seifert	pipe organ	Spišká Nová Ves, SVK	[84]	KF984834
*Talaromyces verruculosus* (Peyronel) Samson, N. Yilmaz, Frisvad & Seifert	cashbox	Wando, KOR	[116]	
*Tapesia* sp.	open-air museum	Riga, LAV	[66]	
*Tetracladium* sp.	archaeological remains	West Greenland, DNK	[73]	
*Thelebolaceae* sp.	historic structures	Whalers Bay and Chilean Station, ATA	[72]	KC514870; KC514867
*Thelebolus globosus* Brumm. & de Hoog	historic structures; timber structures	Whalers Bay, ATA; Port Lockroy, Base E, ATA	[72,86]	KC514885, KC514892; FJ235986
*Thermothelomyces hinnuleus* (Awao & Udagawa) Y. Marín, Stchigel, Guarro & Cano	woodblocks	Yeongju, KOR	[107]	JN639019
*Torula herbarum* (Pers.) Link	concentration camp barracks	Auschwitz, POL	[67]	
*Trichocladium griseum* (Traaen) X. Wei Wang & Houbraken	concentration camp barracks	Auschwitz, POL	[67]	
*Trichoderma* sp.	open-air museum; woodblocks; hunting lodge; artworks; church; historic building, historic building; coffin and wooden boxes; Wawel villa	Riga, LAV; Haeinsa, KOR; Saint Germain-en-Laye; Montefeltro, ITA; Tenaun, CHI; Havana, CUB; Huşi, ROM; Abydos, EGY; Rabka-Zdrój, POL	[66,70,71,74,89,97,98,110,134]	KF638560; MH411225, MH411277
*Trichoderma arenarium* F. Cai, M.Y. Ding & I. S. Druzhinina	hunting lodge	Saint Germain-en-Laye, FRA	[71]	MH411226
*Trichoderma atroviride* P. Karst.	woodblocks	Gonju, KOR	[87]	JX119037
*Trichoderma caerulescens* (Jaklitsch & Voglmayr) Jaklitsch & Voglmayr	woodblocks	Hadong, KOR	[91]	AJ230676
*Trichoderma citrinum* (Pers.) Jaklitsch, W. Gams & Voglmayr	mosque	Cairo, EGY	[82]	
*Trichoderma crassum* Bissett	historic canopies	Los Baños, PHL	[136]	
*Trichoderma koningii* Oudem.	mosque	Cairo, EGY	[82]	
*Trichoderma lixii* (Pat.) P. Chaverri	stored woodblocks	Hapcheo, KOR	[83]	KC008065; JX473719
*Trichoderma longibrachiatum* Rifai	box lid	Abydos, EGY	[110]	MK095972
*Trichoderma reesei* E.G. Simmons	historic canopies	Los Baños, PHL	[136]	
*Trichoderma viridarium* Jaklitsch, Samuels & Voglmayr	hunting lodge	Saint Germain-en-Laye, FRA	[71]	MH411223, MH411224
*Trichoderma viride* Persoon	concentration camp barracks, indoor barracks, Madonna, frames; stored sculptures; statues; cash box; historic canopies	Auschwitz, POL; Auschwitz, POL; Bratislava, SVK; Šid, SRB; Belgrade, SRB; Bratislava, SVK; Wando, KOR; Los Baños, PHL	[67,69,75,76,92,99,116,136]	
*Trichoderma viridescens* (A.S. Horne & H.S. Will.) Jaklitsch & Samuels	hunting lodge	Saint Germain-en-Laye, FRA	[73]	MH411222
*Trichophyton* sp.	funerary boat, church iconostasis	Saqqara, EGY; Boianu Mare, ROM	[80,103]	
*Tricladium terrestre* D. Park.	church	San Juan, CHI	[89]	KF638559
*Tritirachium oryzae* (Vincens) de Hoog	sculptures and painted chair	Havana, CUB	[113]	
*Tritirachium* sp.	historic building	Havana, CUB	[97]	
*Valsa nivea* Fabre	expedition hut	Fort Conger, CAN	[45]	MW033380
*Venustampulla parva* (A.H.S. Br. & G. Sm.) Unter. & Réblová	roof board of stable	Cape Royds, ATA	[123]	GU212410
*Verrucocladosporium dirinae* K. Schub., Aptroot & Crous	stair salt mine	Hallstatt, AUT	[90]	KR081411
*Wettsteinina* sp.	archaeological remains	Wes Greenland, DNK	[73]	
*Xenopolyscytalum pinea* Crous	expedition hut; historic structures	Fort Conger, CAN; Whalers Bay, ATA	[45,72]	MW033381; KC514854, KC514893
*Xenopolyscytalum* sp.	archaeological remains	West Greenland, DNK	[73]	
*Xenopyrenochaetopsis pratorum* (P.R. Johnst. & Boerema) Valenzuela-Lopez, Crous, Stchigel, Guarro & Cano	church	Rilan, CHI	[89]	KF675190
*Zalaria alba* Visagie, Z. Humphries & Seifert	timber structures	East Base, ATA	[86]	FJ235939
*Zalaria obscura* Visagie, Z. Humphries &Seifert	outdoor sculpture	Loreto-Ancona, ITA	[137]	MN480547
*Zasmidium cellare* (Pers.) Fr.	woodblocks	Andong, KOR	[107]	GU322367
*Achanthophysellum fennicum* (Laurilia) Bernicchia & Gorjón	open-air museum	Riga, LAV	[66]	
*Agrocybe cylindracea* (DC.) Maire	stumps	various cities, MKD	[138]	
*Alutaceodontia alutacea* (Fr.) Hjortstam & Ryvarden	open-air museum; historic buildings	Riga, LAV; various cities, LAV	[66,139]	
*Amylocorticiaceae* sp.	historic structures	Whalers Bay and Chilean Station, ATA	[72]	KC514894, KC514895
*Amylocorticiellum molle* (Fr.) Spirin & Zmitr.	hunting cabin interiors, historic structures	Spitsbergen, SJM	[122,140]	
*Antrodia* sp.	open-air museum; roof construction – ceiling beams; church truss structures; woodblocks; woodblocks	Riga, LAV; Various cities, MKD, various cities, CZE; Hapcheon, KOR; Yeongju, KOR	[66,100,107,138,141]	KC951166; AY336785
*Antrodia sinuosa* (Fr.) P. Karst.	open-air museum and sacred buildings; church, traditional houses; ceiling beams and roofs, historic buildings	Riga and Latgale region, LAV; Amărăşti, ROM; Kizhi, RUS; Various cities, MKD, Kizhi, RUS	[66,81,130,138,139,142]	
*Antrodia xantha* (Fr.) Ryvarden	open-air museum; historic buildings	Riga, LAV; various cities, LAV	[66,139]	
*Athelia decipiens* (Höhn. & Litsch.) J. Erikss.	old guest house porch	various cities, MKD	[138]	
*Athelia epiphylia* (Höhn. & Litsch.) J. Erikss.	open-air museum; roof, historic buildings	Riga, LAV; Macedonia, MDN; various cities, LAV	[66,138,139]	
*Athelia neuhofii* (Bres.) Donk	open-air museum and sacred building, wall roof construction	Riga and Latgale region, LAV; Macedonia, MDN;	[66,138]	
*Athelia pyriformis* (M. P. Christ) Jülich	roof construction	various cities, MKD	[138]	
*Athelia* sp.	open-air museum and sacred buildings; roof and walls; historic buildings	Riga and Latgale region, LAV; Various cities, MKD; various cities, LAV	[66,138,139]	
*Aurantiporus albidus* Rajchenb. & Cwielong	church	Tenaun, CHI	[89]	KF638544
*Auricularia auricola-judae* (Bull.) J. Schröt.	fence and benches	various cities, MKD	[138]	
*Auricularia mesenterica* (Dicks.) Pers.	porch beams and chair; historcal buildings	various cities, MKD; various cities, LAV	[138,139]	
*Bjerkandera adusta* (Willd.) P. Karst.	woodblocks; churches, woodblocks, churches and monasteries; roof and stairs; historic buildings	Uiwan, KOR; Achao and Quinchao, CHI; Ulsan and Hapcheon, KOR; various cities, MDA; various cities, MKD; various cities, LAV	[87,89,100,135,139]	KF638514, KF638527, KF475891; FJ810147; KF313125
*Botryobasidium candicans* J. Erikss.	open-air museum; historic buildings	Riga, LAV; various cities, LAV	[66,139]	
*Botryobasidium laeve* (J. Erikss.) Parmasto	open-air museum	Riga, LAV	[66]	
*Botryobasidium obtusisporum* Johan Erikson	roof constructions- stairs	various cities, MKD	[138]	
*Botryobasidium subcoronatum* (Höhn. Litsch.) Donk	open-air museum	Riga, LAV	[66]	
*Botryobasidium vagum* (Berk. & M. A. Curtis) D. P. Rogers	open-air museum; church	Riga, LAV; Certaldo, ITA	[66,125]	
*Byssomerulius corium* (Pers.) Parmasto	historic buildings	various cities, LAV	[139]	
*Candolleomyces* sp.	woodblocks	Andong, KOR	[107]	KU324797
*Cantharellales* sp.	historic structures	Whalers Bay, ATA	[72]	KZ514909
*Ceraceomyces sublaevis* (Bres.) Jülich	sacred buildings	Latgale region, LAV	[66]	
*Cerinosterus* sp.	timber structures	Horseshoe Island, ATA	[86]	FJ235994
*Ceriporia purpurea* (Fr.) Komarova	historic buildings	various cities, LAV	[139]	
*Ceriporia reticulata* (Hoffm.) Domański	historic buildings	various cities, LAV	[139]	
*Ceriporiopsis anereina* (Sommerf. Ff.) Dom	roof construction	Various cities, MKD	[138]	
*Ceriporiopsis excelsa* (S. Lundell) Parmasto	beams of the bell tower	Various cities, MKD	[138]	
*Ceriporiopsis resinascens* (Romell) Domanski	old guest house	Various cities, MKD	[138]	
*Ceriporiopsis* sp.	old guest house	Various cities, MKD	[138]	
*Cerrena unicolor* (Bull.) Murrill	temple surfaces	Haeinsa, KOR	[132]	
*Chondrostereum purpureum* (Pers.) Pouzar	roof construction	Various cities, MKD	[138]	
*Clitopilus baronii* Consiglio & Setti	church	Castro, CHI	[89]	KF638517
*Collybiopsis subpruinosa* (Murrill) R.H. Petersen	church	Achao, CHI	[89]	KF638511
*Coniophora* sp.	historic canopies; church truss structures	Los Baños, PHL; various cities, CZE	[136,141]	
*Coniophora arida* (Fr.) Karst	beams in basement, historic buildings	various cities, MKD; various cities, LAV	[138,139]	
*Coniophora olivacea* (Fr.) Karst	roof construction	various cities, MKD	[138]	
*Coniophora puteana* (Schumach.) P. Karst.	wall board and floor; historic structure; church; churches; timber structures; traditional houses; churches and monasteries; guest house; historic buildings; hunting cabin interiors; protected buildings Auram Iancu Memorial house	Riga and Latgale region LAV; Whalers Bay, ATA; San Juan, CHI; various cities, ROM; Spitsbergen, SJM; Kizhi, RUS; various cities, MDA; various cities, MKD; various cities, LAV; various sites, SJM; Kizhi, RUS; Vidra de Sus, ROM;	[66,72,89,105,122,130,135,138,139,140,142,143]	KF638536; KC514900
*Coprinellus* aff. *xanthothrix* Romagn.	church walls	Nicula, ROM	[88]	HF543673
*Coprinellus micaceus* (Bull.) Vilgalys, Hopple & Jacq. Johnson	historic structures, old beams	Chilean Station, ATA; various cities, MKD	[72,138]	KC514901
*Coprinellus radians* (Desm.) Vilgalys, Hopple & Jacq. Johnson	hunting lodge, stored woodblocks	Germaine-en-Laye, FRA; Hapcheo, KOR; Saint	[71,83]	FJ462761; MG920351, MN071393, MN071394
*Coriolopsis galica* (Fr.) Ryvarden	doors	various cities, MKD	[138]	
*Corticiaceae* sp.	indoor barracks, historic buildings, hunting cabin, protected buildings	Auschwitz, POL; Whalers Bay, ATA; Spitsbergen, SJM; various sites, SJM	[69,72,122,140]	
*Corticiales* sp.	church	Quinchao, CHI	[89]	KF638528
*Corticium roseum* Pers.	historic buildings	various cities, LAV	[139]	
*Crepidotus cesatii* (Rabenh.) Sacc.	beams	various cities, MKD	[138]	
*Crepidotus mollis* (Schaeff.) Staude	historic buildings	various cities, LAV	[139]	
*Crustoderma drynum* (Berk. & M. A. Curtis) Parmasto	open-air museum	Riga, LAV	[66]	
*Cryptococcus* sp.	pirogue	Tianjin, CHN	[94]	
*Cuniculitremaceae* sp.	hunting lodge	Saint Germaine-en-Laye, FRA	[71]	MG920348
*Cylindrobasidium evolvens* S. (Fr.) Fr.	tower bell beams; hunting cabin	various cities, MKD; Spitsbergen, SJM	[122,136]	
*Cylindrobasidium laeve* (Pers.) Chamuris	concentration camp barracks	Auschwitz, POL	[67]	
*Cystobasdium* sp.	historic structures, timber structures	Cape Evans, and Discovery hut, ATA; Cape Evans, Cape Royds, Discovery hut, Port Lockroy, Detaille Island, ATA	[85,86]	DQ317357, DQ317365; FJ236004
*Cystobasidium laryngis* (Reiersöl) Yurkov, Kachalkin, H.M. Daniel, M. Groenew., Libkind, V. de García, Zalar, Gouliam., Boekhout & Begerow	timber structures	Hope Bay, Horseshoe Island, ATA	[86]	FJ236002, FJ236005
*Cystobasidium psychroaquaticum* Yurkov, Kachalkin, H.M. Daniel, M. Groenew., Libkind, V. de García, Zalar, Gouliam., Boekhout & Begerow	timber structures	Base E, ATA	[86]	FJ235993
*Cystobasidium raffinophilum* Q.M. Wang, F.Y. Bai & A.H. Li	timber structures	East Base, ATA	[86]	FJ236003
*Cystobasidium slooffiae* (E.K. Novák & Vörös-Felkai) Yurkov, Kachalkin, H.M. Daniel, M. Groenew., Libkind, V. de García, Zalar, Gouliam., Boekhout & Begerow	icons	Nicula, ROM	[88]	JQ993376
*Dacrymyces stillatus* Nees.	churches, church; churches and monasteries; old guest house, churches	Quinchao and Nercón, CHI; various cities, ROM; various cities, MDA; various cities, MKD Vrancea County, ROM	[89,105,135,138,144]	KF638525, KF638533
*Dacryobolus sudans* (Alb. & Shwein.) Fr.	open-air museum, hunting cabin, historic buildings	Riga, LAV; Spitsbergen, SJM; various cities, LAV	[63,118,135];	
*Daedalea quercina* (L.) Pers.	churches and monasteries	various cities, MDA	[134]	
*Dentipellis fragilis* (Pers.) Donk	woodblocks	Yeongju, KOR	[107]	AF334911
*Ditiola radicata* (Alb. & Schwein.) Fr.	hunting cabin	Spitsbergen, SJM	[122]	
*Donkioporia expansa* (Desm.) Kotl. & Pouzar	church	Montalcino, ITA	[125]	
*Efibula tuberculata* (P. Karst.) Zmitr. & Spirin	roof construction church in open dome; historic buildings	various cities, MKD; various cities, LAV	[138,139]	
*Exidia glandulosa* (Bull.) Fr.	indoor staircase-roof-beams	various cities, MKD	[139]	
*Exidiopsis calcea* (Pers.) K. Wells	open-air museum; historic buildings	Riga, LAV; various cities, LAV	[66,139]	
*Exidiopsis* sp.	gateway and boards	various cities, MKD	[138]	
*Exobasidiales* sp.	hunting lodge	Saint Germaine-en-Laye, FRA	[73]	MN071391, MN071392
*Fibroporia vaillantii* (DC.) Parmastro	open-air museum, concentration camp barracks, indoor barracks, church, churches, traditional houses, churches and monasteries, historic buildings, historic timber structures, historic house, churches	Riga, LAV; Auschwitz, POL; Auschwitz, POL; Huşi, ROM; various cities, ROM; Kizhi, RUS; various cities, MDA; various cities, LAV; Kizhi, RUS; Vrancea county, ROM	[66,67,69,98,105,130,135,139,142,144]	
*Fomitiporella* sp.	churches	Achao, CHI	[89]	KF638515
*Fomitopsis pinicola* (Swartz) P. Karsten	historic buildings	various cities, LAV	[139]	
*Fomitopsis rosea* (Alb. & Schwein.) P. Karst.	sacred building; wooden churches	Latgale region, LAV; various cities, ROM	[66,105]	
*Funalia gallica* Fr. Bondartsev & Singer	roof construction	various cities, MKD	[138]	
*Fuscoporia contigua* (Pers.) G. Cunn.	churches; churches and monasteries; churches	various cities, ROM; various cities, MDA; Vrancea county, ROM	[105,135,144]	
*Galerina* sp.	boards and poles	various cities, MKD	[138]	
*Galerina hypnorum* (Shrank) Kühner	open-air museum	Riga, LAV	[66]	
*Ganoderma adspersum* (Schulzer) Donk	watch tower	various cities, MKD	[138]	
*Gloeocystidiellum convolvens* P. Karst. Donk	beams	various cities, MKD	[138]	
*Gloeocystidiellum luridum* (Bres.) Boidin	gate and planks, historic buildings	various cities, MKD; various cities, LAV	[138,139]	
*Gloeocystidiellum porosum* (Berk. & M. A. Curtis) Donk.	beams and interior stairs	various cities, MKD	[138]	
*Gloeophyllum abietinum* (Bull.) P. Karst.	open-air museum; churches and monasteries; ceiling, roof and tower beams; historic buildings, churches	Riga LAV; various cities, MDA; various cities, MKD; various cities, LAV; Vrancea county, ROM	[66,135,138,139,144]	
*Gloeophyllum sepiarium* (Wulfen) P. Karst.	open-air museum, wooden churches, hunting cabin; churches and monasteries; fence, porch and roofs; historic buildings, protected buildings, traditional houses, churches	Riga LAV; various cities, ROM; Spitsbergen, SJM; various cities, MDA; various cities, MKD; various cities, LAV; various sites, SJM; Kizhi, RUS; Vrancea county, ROM	[66,105,122,135,138,139,140,142,144]	
*Gloeophyllum* sp.	church truss structures	various cities, CZE	[141]	
*Gloeophyllum trabeum* (Pers.) Murrill	open-air museum; wood support	Riga LAV; various cities, MKD	[66,138]	
*Gloiothele citrina* (Pers.) Ginns & G. W. Freeman	open-air museum; wooden churches	Riga, LAV; various cities, ROM	[66,105]	
*Habeloma* sp.	woodblocks	Andong, KOR; Seoul, KOR	[101,107]	DQ465339; AB084593
*Hapalopilus nidulans* (Fr.) P. Karst.	roof construction	various cities, MKD	[138]	
*Haplotrichum capitatum* (Link) Link	open-air museum	Riga, LAV	[66]	
*Hymenochaetaceae* sp.	locomotive turntable	La Plata, ARG	[93]	
Hymenochaetales *incertae sedis*	woodblocks	Andong, KOR	[107]	AF082856
*Hymenochaete fuliginosa* (Pers.) Lév.	roof beams	various cities, MKD	[138]	
*Hymenochaete rubiginosa* (Dicks.) Lév.	churches and monastic ensembles	various cities, MDA	[135]	
*Hyphoderma argillaceum* (Bres.) Donk	sacred buildings	Latgale region, LAV	[66]	
*Hyphoderma obtusiforme* J. Erikss & Å.Strid	open-air museum, roof old guest house	Riga, LAV; various cities, MKD	[66,138]	
*Hyphoderma obtusum* J. Erikss.	historic buildings	various cities, LAV	[139]	
*Hyphoderma occidentale* (D.P. Rogers) Boidin & Gilles	sacred buildings	Latgale region, LAV	[66]	
*Hyphoderma praetermissum* (P. Karst.) J. Erikss & Å.Strid	open-air museum, loghouse wall, historic buildings	Riga, LAV; Various cities, MKD, various cities, LAV	[66,138,139]	
*Hyphoderma puberum* (Fr.) Walir.	open-air museum, churches; staircase and bell tower; historic buildings	Riga, LAV; various cities, ROM; various cities, MKD; various cities, LAV	[66,105,138,139]	
*Hyphoderma setigerum* (Fr.) Donk	hunting cabin, roof construction	Spitsbergen, SJM; various cities, MKD;	[122,138]	
*Hyphoderma tenue* (Pat.) Donk	protected buildings	various sites, SJM	[140]	
*Hyphodermella* sp.	church; woodblocks	Achao, CHI; Sokcho, KOR	[89,101]	KF638510; **JN940190**
*Hyphodontia arguta* (Fr.) J. Erikss.	churches; old guest house	various cities, ROM; various cities, MKD	[105,138]	
*Hyphodontia microspora* J. Erikss. & Hyortst	beams, stairs and chairs	various cities, MKD	[138]	
*Hyphodontia pallidula* (Bres.) J. Erikss.	porches and fences	various cities, MKD	[138]	
*Hyphodontia* sp.	roofs	various cities, MKD	[138]	
*Hypholoma fasciculare* (Huds.) P. Kumm.	benches, historic buildings	various cities, MKD, various cities, LAV	[138,139]	
*Hypochnicium bombycinum* (Sommerf.) J. Erikss.	historic buildings	various cities, LAV	[139]	
*Hypochnicium punctulatum* (Cooke) J. Erikss.	open-air museum	Riga, LAV	[66]	
*Irpex lacteus* (Fr.) Fr.	chairs, benches and vaults	various cities, MKD	[138]	
*Irpex* sp.	woodblocks	Sokcho, KOR	[101]	KP135224
*Jaapia argillacea* Bres.	historic structure	Whalers Bay, ATA	[72]	KC514904
*Junghuhnia nitida* (Pers.) Ryvarden	gates	various cities, MKD	[138]	
*Kneiffia subalutacea* (P. Karst.) Bres.	beams and stairs	various cities, MKD	[138]	
*Lacnocladiaceae* sp.	woodblocks	Sokcho, KOR	[101]	**U59085**
*Laetiporus sulphureus* (Bull.) Murrill	church	Tenaun, CHI	[89]	KC514814
*Lentinus* sp.	church truss structures	various cities, CZE	[141]	
*Leucogyrophana pseudomollusca* (Parmasto) Parm.	beams	various cities, MKD	[138]	
*Lopharia spedicea* (Pers.) Boidin	roofs	various cities, MKD	[138]	
*Lyomyces crustosus* (Pers.) P. Karst.	roof and porches, historic buildings	various cities, MKD; various cities, LAV	[138,139]	
*Lyomyces sambuci* (Pers.) P. Karst.	beams, stairs and chairs	various cities, MKD	[138]	
*Marasmius torquescens* Quél.	bell tower support	various cities, MKD	[138]	
Microstromatales *incertae sedis*	woodblocks	Andong, KOR	[107]	HM595622
*Mycena galericulata* (Scop.) Gray	sacred buildings	Latgale region, LAV	[66]	
*Mycena silvae-nigrae* Maas Geest.	open-air museum	Riga, LAV	[66]	
*Mycena* sp.	sacred buildings, balcony beam	Latgale region, LAV; various cities, MKD	[66,138]	
*Mycena stipata* Maas Geest. & Schwöbel	open-air museum	Riga, LAV	[66]	
*Mycoacia livida* (Pers.) Zmitr.	salt mine stair	Hallstatt, AUT	[90]	KR081412
*Naganishia albidosimilis* (Vishniac & Kurtzman) Xin Zhan Liu, F.Y. Bai, M. Groenew. & Boekhout	historic structures	Cape Evans, Cape Royds, Allan Hill, ATA	[85]	DQ317387
*Neoantrodia serialis* (Fr.) Audet	hunting cabin, heritage buildings, protected buildings	Spitsberger, SJM; various cities, LAV; various sites, SJM	[122,139,140]	
*Neolentinus lepideus* (Fr.) Redhead & Ginns	porches of traditional houses	Kizhi, RUS	[142]	
*Odontia fibrosa* (Berk. & M.A. Curtis) Kõljalg,	old guest house	various cities, MKD	[138]	
*Peniophora cinerea* (Pers.) Cooke	old guest house, historic buildings	various cities, MKD; various cities, LAV	[138,139]	
*Peniophora incarnata* (Pers.) P. Karst.	Old guest house, historic buildings	various cities, MKD; various cities, LAV	[138,139]	
*Peniophora pithya* (Pers.) J. Erikss.	Porch roof	various cities, MKD	[138]	
*Peniophorella praetermissa* (P. Karst.) K.H. Larss	church	Calen and Tenaun, CHI	[89]	KF638519, KF638545
*Peniophorella pubera* (Fr.) P. Karst.	sacred buildings	Latgale region, LAV	[66]	
*Phaeolus schweinitzii* (Fr.) Pat.	Historic buildings	various cities, LAV	[139]	
*Phanerochaete calotricha* (Karst.) Erikss & Ryvarden	roofs	various cities, MKD	[138]	
*Phanerochaete laevis* (Fr.) J. Erikss & Ryvarden	beams and boards	various cities, MKD	[138]	
*Phanerochaete sordida* (Karst.) Erikss & Ryvarden	sacred buildings, church, lodge roof	Latgale region, LAV; Certaldo, ITA; various cities, MKD;	[66,125,138]	
*Phanerochaete velutina* (DC.) P. Karst.	Roof construction and benches	various cities, MKD	[138]	
*Phellinus chrysoloma* (Fr.) Donk	sacred buildings	Latgale region, LAV	[66]	
*Phellinus cryptarum* Quél.	Churches	various cities, ROM	[105]	
*Phellinus punctatus* (P. Kurst.) Pilát	roof construction and fence	various cities, MKD	[138]	
*Phlebia livida* (Pers.) Bres.	Old guest house	various cities, MKD	[138]	
*Phlebia rufa* (Pers.) M.P. Christ	church	Quinchao and San Juan, CHI	[89]	KF638531, KF638537
*Phlebia segregata* (Bourdt & Galzin) Parmasto	old guest house	various cities, MKD	[138]	
*Phlebiopsis gigantea* (Fr.) Jülich	open-air museum, stored sculpture, historic buildings	Riga, LAV; Coimbra, PRT; various cities, LAV	[66,120,139]	
*Phlebiopsis roumengueri* (Bresad.) Jülich & Stalp	old guest house	various cities, MKD	[138]	
*Phlebiopsis* sp.	Woodblocks, woodblocks, church truss structures, traditional house	Uiwang, KOR; Ulsan, KOR; various cities, CZE; Kizhi, RUS	[87,100,141,142]	FJ791151; HQ331053
*Pleurotus dryinus* (Pers.) P. Kumm	bell tower	various cities, MKD	[138]	
*Pluteus phlebophorus* (Ditmar)	roof	various cities, MKD	[138]	
*Pluteus semibulbosus* (Lasch) Quél.	Historic buildings	various cities, LAV	[139]	
*Polyporales* sp.	Churches, structures saltpeter works	Calen and Quinchao, CHI; Humbertone and Santa Laura, CHI	[89,121]	KF638518, KF638526; FN812727
*Postia caesia* (Schrad.) P. Karst.	sacred buildings	Latgale region, LAV	[66]	
*Postia fragilis* (Fr.) Jülich	open-air museum	Riga, LAV	[66]	
*Postia guttulata* (Peck) Jülich	open-air museum	Riga, LAV	[66]	
*Postia stiptica* (Pers.) Julich	historic buildings	various cities, LAV	[139]	
*Postia subcaesia* (David) Jül	old guest house	various cities, MKD	[138]	
*Postia wakefieldiae* (Kotl. & Pouzar) Pegler & E.M. Saunders	church	Calen, CHI	[89]	KF638524
*Postiaceae* sp.	Historic structures	Whalers Bay, ATA	[72]	KC514907
*Psathyrellaceae* sp.	structures of saltpeter works	Humbertone and Santa Laura, CHI	[121]	JF681946
*Pycnoporellus fulgens* (Fr.) Donk	historic buildings	various cities, LAV	[139]	
*Radulomyces confluens* (Fr.) M. P. Christ.	churches and monasteries; old guest house	various cities, MDA; various cities, MKD	[135,139]	
*Resinicium bicolor* (Alb. & Schwein.) Parmasto	open-air museum, historic buildings	Riga, LAV; various cities, LAV	[66,139]	
*Resinoporia sordida* (Ryvarden & Gilb.) Audet	historic buildings	various cities, LAV	[139]	
*Resupinatus applicatus* (Batsch: Fr.) Gray	old guest house roof	various cities, MKD	[138]	
*Rhizochaete filamentosa* (Berk. & M.A. Curtis) Gresl., Nakasone & Rajchenb	old guest house	various cities, MKD	[138]	
*Rhizochaete radicata* (Henn.) Gresl., Nakasone & Rajchenb	beams and boards	various cities, MKD	[138]	
*Rhizoctonia solani* J.G. Kühn	temple surfaces	Haeinsa, KOR	[132]	
*Rhodotorula* sp.	artworks, pirogue	Montefeltro, ITA; Tianjin, CHN	[74,94]	
*Roseograndinia* sp.	churches	Castro, CHI	[89]	KF638516
*Schizophyllum commune* Fr.	open-air museum, indoor church surface, woodblocks, churches and monasteries, historic buildings, churches	Riga, LAV; Bucharest, ROM; Sokcho, KOR; various cities, MDA; various cities, LAV; Vrancea county, ROM	[66,79,101,134,139,144]	
*Schizopora paradoxa* (Schrad.) Donk	churches, old guest house roof, historic buildings;	various, ROM; various cities, MKD; various cities, LAV	[105,138,139]	
*Scytinostroma* cft. *odoratum* (Fr.) Donk	historic buildings	various cities, LAV	[139]	
*Serpula lacrymans* (Wulfen) P. Karst.	sacred buildings, concentration camp barracks, hunting lodge, historic structures, traditional houses; gilded ceiling decoration, churches, church floor boards; historic timber structures; historic building, historic timber structures; Auram Iancu memorial house; ancient church	Latgale region, LAV; Auschwitz, POL; Saint Germain-en-Laye, FRA; Huşi, ROM; Kizhi, RUS; Averio, PRT; Cerviceşti and Agafton, MDA; various cities, MKD; various cities, LAV; Huşi, ROM; Kizhi, RUS; Vidra de Sus, ROM; Horodniceni, ROM	[66,67,73,97,130,131,135,138,139,142,143,145]	JF734883
*Serpula* sp.	church truss structures	various cities, CZE	[141]	
*Sistotrema brinkmannii* (Bres.) J. Erikss.	historic structures; timber structures; church, castle, protected buildings	Chilean station, ATA; Hope Bay, Wordie House, Detaille Island, ATA; Quinchao, CHI Este, ITA; various sites, SJM	[72,86,89,125,140]	KC514823; KC514908; FJ236006
*Sistotrema efibulatum* J. Erikss.	old guest house	various cities, MKD	[138]	
*Skeletocutis carneogrisea* A. David	historic buildings	various cities, LAV	[139]	
*Skeletocutis percandida* (Malencon & Bertault) J. Keller	old guest house	various cities, MKD	[138]	
*Sporobolomyces salmonicolor* B. Fisch. & Brebeck ex Kluyver & C.B. Niel	timber structures	Base E, ATA	[86]	FJ236007
*Sporobolomyces* sp.	historic structures	Discovery Hut, ATA	[85]	DQ317366
*Steccherinum bourdotii* Saliba & A. David	beams and boards	various cities, MKD	[138]	
*Stereum armeniacum* Boidin & Gilles	church	Calen, CHI	[89]	KF638520
*Stereum hirsutum* (Willd.) Pers.	church, churches and monasteries, chairs, benches and barrel vaults, churches	Achao, CHI; various cities, Modavia; Various cities, MKD; Vrancea county, ROM	[89,135,138,144]	
*Stereum sanguinolentum* (Alb. & Schwein.) Fr.	open-air museum, historic buildings	Riga, LAV; various cities, LAV	[66,139]	
*Stereum* sp.	woodblocks, church truss structures	Sokcho, KOR; various cities, CZE;	[101,140]	KU574826
*Sterigmatomyces halophilus* Fell	salt mine stair	Hallstatt, AUT	[90];	KR081406
Strophariaceae sp.	historic structures	Whalers Bay, Chilean Station, ATA	[72]	KC514905, KC514906
*Symmetrospora symmetrica* (F.Y. Bai & Q.M. Wang) Q.M. Wang, F.Y. Bai, M. Groenew. & Boekhout	historic structures	Cape Evans, ATA	[85]	DQ317384
*Tapinella panuoides* (Batsch) E. J. Gilbert	historic timber structures, roof construction old guest house; historic buildings,	Kizhi, RUS; Various cities, MKD; various cities, LAV	[130,138,139]	
*Tomentella* cft. *cinerascens* (P. Karst.) Höhn. & Lisch.	open-air museum	Riga, LAV	[66]	
*Tomentella ferruginella* Pers. Ex Pat.	timber structures	various cities, MKD	[138]	
*Tomentella terrestris* (Berk. & Broome) M. J. Larsen	open-air museum	Riga, LAV	[66]	
*Topinella panuoides* (Fr.)E.-J. Gilbert	open-air museum	Riga, LAV	[66]	
*Trametes palisotii* (Fr.) Imazeki	church	Rome, ITA	[125]	
*Trametes* sp.	church truss structures	various cities, CZE	[141]	
*Trametes trogii* (Berk.)	benches and bell tower	various cities, MKD	[138]	
*Trametes versicolor* (L.) Lloyd	churches, woodblocks, churches and monastic ensembles, chair and benches	Calen, Tenaun, and San Juan, CHI; Andong, KOR; various cities, MDA; Various cities, MKD	[88,107,135,138]	KF638522, KF638535, KF638540; AY309017
*Trechispora farinacea* (Pers.: Fr.) Liberta	open-air museum and sacred buildings, old guest house	Riga and Latgale region, LAV; various cities, MKD,	[66,138]	
*Trechispora* sp.	old guest house	various cities, MKD	[138]	
*Tremella mesenterica* (Schaeff.) Retz.	old guest house	various cities, MKD	[138]	
Tremellales sp.	archaeological remains	West Greenland, DNK	[73]	
*Trichaptum abietinum* (Pers. ex J.F. Gmel.) Ryvarden	historic buildings	various cities, LAV	[139]	
*Trichaptum fusco-violaceum* (Ehrenb.) Ryvarden	open-air museum	Riga, LAV	[66]	
*Tubulicrinis calothrix* (Pat.) Donk	sacred buildings	Latgale region, LAV	[66]	
*Tubulicrinis glebulosum* (Fr.) Donk	open-air museum; roof and vault beams	Riga, LAV; various cities, MKD	[66,138]	
*Tubulicrinis medius* (Bourdot & Galzin) Oberw.	open-air museum; roof and vault beams	Riga, LAV; various cities, MKD	[66,138]	
*Tyromyces* cft. *tephroleucus* (Fr.) Donk	old guest house roof	various cities, MKD	[138]	
*Tyromyces* sp.	expedition hut	Fort Conger, CAN	[45]	MW033378, MW033379
*Veluticeps abietina* (Pers.) Hjortstam & Tellería	hunting cabin	Spitsbergen, SJM	[122]	
*Vishniacozyma carnescens* (Verona & Luchetti) Xin Zhan Liu, F.Y. Bai, M. Groenew. & Boekhout	historic structures	Cape Evans, and Cape Royds, ATA	[85]	DQ317388
*Vishniacozyma victoriae* (M.J. Montes, Belloch, Galiana, M.D. García, C. Andrés, S. Ferrer, Torr.-Rodr. & J. Guinea) Xin Zhan Liu, F.Y. Bai, M. Groenew. & Boekhout	historic structures; timber structures	Cape Evans, Cape Royds, and Discovery Hut, ATA; Hope Bay, Port Lockroy, Detaille Island, Horseshoe Island, ATA	[85,86]	DQ317363; FJ236000
*Wallemia muriae* (Kickx) Zalar & de Hoog	salt mine stair	Hallstatt, AUT	[90]	KR081403
*Xylodon asper* (Fr.) Hjortstam & Ryvarden	open-air museum and sacred buildings, inner door historic buildings	Riga and Latgale region, LAV; Various cities, MKD, various cities, LAV	[66,138,139]	
*Xylodon brevisetus* (P. Karst.) Hjortstam & Ryvarden	sacred buildings, churches; churches and monasteries; historic buildings; churches	Latgale region, LAV; various cities, ROM; various cities, MDA; various cities, LAV; Vrancea county, ROM	[66,105,135,139,144]	
*Xylodon detriticus* (Bourdot) K.H. Larss., Viner & Spirin, in Viner, Spirin, Zíbarová & Larsson	open-air museum	Riga, LAV	[66]	
*Xylodon taiwanianus* (Sheng H. Wu) Hjortstam & Ryvarden	historic buildings	Whalers Bay, ATA	[72]	KC514902
*Absidia glauca* Hagem	indoor barracks	Auschwitz, POL	[69]	
*Entomortierella* sp.	historic structures	Whalers Bay, ATA	[72]	KC514911
*Lichtheimia corymbifera* (Cohn) Vuill.	sculptures	Belgrade, SRB	[92]	
*Linnemannia amoeboidea* (Gams) Vandepol & Bonito	expedition hut	Fort Conger, CAN	[47]	MW033353
*Linnemannia gamsii* (Milko) Vandepol & Bonito	expedition hut	Fort Conger, CAN	[47]	MW033354
*Linnemannia hyalina* (Gams) Vandepol & Bonito	expedition hut	Fort Conger, CAN	[47]	MW033355
*Linnemannia* sp.	historic structures	Whalers Bay, ATA	[72]	KC514913
*Mortierella alpina* Peyronel	historic structures	Whalers Bay, Chilean station, ATA	[72]	KC514910
*Mortierella polycephala* Coem.	timber structures	East Base, ATA	[86]	FJ236011
*Mortierella* sp.	archaeological remains	West Greenland, DNK	[73]	
*Mortierellales* sp.	historic structures	Whalers Bay, Chilean station, ATA	[72]	KC514912
*Mucor hiemalis* Wehmer	expedition hut	Fort Conger, CAN	[45,71,73]	MW033356
*Mucor* sp.	hunting lodge, archaeological remains, artworks, locomotive turntable, historic building, gilded woodcarving	Saint Germain-en-Laye, FRA; West Greenland, DNK; Montefeltro, ITA; La Plata, ARG; Havana, CUB; Evora, PRT	[71,73,74,93,97,129]	
*Mucor circinelloides* Tiegh.	indoor church surface	Juliţa, ROM	[79]	
*Mucoraceae* sp.	timber structures	East Base, ATA	[86]	FJ236009
*Mycotypha microspora* Fenner	frames	Šid, SRB	[76]	
*Rhizomucor pusillus* (Lindt) Schipper	indoor barracks	Auschwitz, POL	[69]	
*Rhizopus* sp.	hunting lodge, artworks; locomotive turntable; historic building, church; historic canopies	Saint Germain-en-Laye, FRA; Montefeltro, ITA; La Plata, ARG; Havana, CUB; Spălanca, ROM; Los Baños, PHL	[71,74,93,97,105,136]	
*Rhizopus stolonifer* (Ehrenb.) Vuill.	indoor barracks, Madonna, concentration camp, stored statues; frames; indoor church surfaces sculptures	Auschwitz, POL; Belgrade, SRB; Šid, SRB; Bratislava, SVK; Bârzava, Bucharest, and Troaş-Săvârşin, ROM; Bratislava, SVK	[69,75,76,79,92,99]	
*Syncephalastrum* sp.	historic building	Havana, CUB	[97]	
*Syncephalastrum racemosum* Cohn ex J. Schröt.	artefacts from museum, frame, woodblocks	Belgrade, SRB; Cairo, EGY; Ulsan, KOR	[80,92,100]	KC117254
*Umbelopsis* sp.	archaeological remains; hunting lodge	West Greenland, DNK; Saint Germain-en-Laye, FRA	[71,73]	MH411230

ARG: Argentina, ATA: Antarctica, AUT: Austria, CAN: Canada, CHE: Switzerland, CHI: Chile, CHN: China, CUB: Cuba, CZE: Czechia, DNK: Denmark (Greenland), EGY: Egypt, FRA: France, GER: Germany, HRV: Croatia, IDN: Indonesia, ITA: Italy, JOR: Jordan, KOR: South Korea, LAV: Latvia, MAR: Morocco, MDA: Moldova, MKD: North Macedonia, POL: Poland, PRT: Portugal, RMN: Romania, RUS: Russia, SRB: Serbia, SJM: Svalbard, SVK: Slovakia, SVN: Slovenia, PHL: Philippines.

**Table 3:** Lignocellulolytic enzymatic activities recorded for a selection of species whose presence has been documented in terrestrial WCH. Evidence of cellulolytic activities coming from tests performed using filter paper, cellulose and its soluble derivates have been merged in the column ‘Cellulase’. Detailed recipes of media used are reported in Appendix A.

	Cellulase	β-Glucosidase	Xylanase	Ligninase	Laccase	Lignin Peroxidase	Mn Peroxidase	Phenol Oxidase
*Alternaria alternata*	[195,203,204]				[201]	[199]	[199]	[204]
*Alternaria angustiovoidea*	[83,84]		[83]	[83]			[84]	
*Alternaria chartarum*	[204]							[204]
*Alternaria pogostemonis*	[83]		[83]					
*Alternaria tenuissima*	[125]							
*Alternaria* sp.	[125]							
*Apiospora arundinis*				[83]				
*Apiospora sacchari*				[83]				
*Apiospora sphaerosperma*	[102]						[102]	
*Aspergillus amstelodami*							[102]	
*Aspergillus candidus*	[203]							
*Aspergillus chevalieri*	[84]				[84]	[84]	[84]	
*Aspergillus cristatus*	[84]						[84]	
*Aspergillus fischeri*							[102]	
*Aspergillus flavipes*	[203]							
*Aspergillus flavus*	[203]		[205]		[199]	[199]	[102,199]	
*Aspergillus fumigatus*			[205]		[199]	[199]	[199]	
*Aspergillus niger*	[203]		[205]			[199]	[102,199]	
*Aspergillus oerlinghausenensis*				[83]				
*Aspergillus ochraceus*			[205]					
*Aspergillus sydowii*	[84]		[132,205]		[84]		[84]	
*Aspergillus terreus*	[75]	[97]			[75]	[75]	[102]	
*Aspergillus ustus*	[102]				[102]		[102]	
*Aspergillus versicolor*	[84,206]		[132]		[84]		[84]	
*Aureobasidium pullulans*	[207]							
*Beauveria bassiana* f							[102]	
*Bjerkandera adusta*	[206]							
*Cadophora malorum*	[208]							
*Chaetomium elatum*	[102,206]				[102]		[102]	
*Chaetomium globosum*	[75]				[75]		[102]	
*Cladosporium cladosporioides*	[75,128,195]				[75]		[102]	
*Cladosporium herbarum*	[204]							
*Cladosporium sphaerospermum*	[204]							
*Cladosporium perangustum*	[84]						[80]	
*Cladosporium pseudocladosporioides*	[195]							
*Cladosporium* sp.	[195]							
*Cladosporium sphaerospermum*	[195]							
*Coniochaeta hoffmannii*	[197]	[197]	[197]		[197]			
*Coprinellus radians*	[83]		[83]	[83]				
*Debaryomyces hansenii*	[192]							
*Epicoccum nigrum*	[84]		[83]	[83]			[84]	
*Epicoccum* sp.			[83]	[83]				
*Exophiala xenobiotica*	[192,197]							
*Fusarium oxysporum*	[203]							
*Fusarium solani*	[200]				[200]			
*Fusarium annulatum*				[83]				
*Fusarium reticulatum*			[83]	[83]				
*Neurospora crassa*	[203]							
*Neurospora sitophila*	[203]							
*Nigrospora oryzae*	[203]							
*Paecilomyces maximus*	[84]						[84]	
*Paecilomyces variotii*	[203]							
*Paraphaeosphaeria* sp.	[83]		[83]	[83]				
*Penicillium brevicompactum*	[193,204]							
*Penicillium chrysogenum*	[75,128,193]				[75]		[102]	
*Penicillium citreonigrum*	[193]		[132]					
*Penicillium citrinum*	[193]							
*Penicillium commune*	[128]							
*Penicillium crustosum*	[84,128]				[80]	[80]	[80]	
*Penicillium digitatum*	[193]							
*Penicillium expansum*	[102,128,193,208]						[102]	
*Penicillium glabrum*	[193]							
*Penicillium granulatum*	[128]							
*Penicillium herquei*	[75]						[102]	
*Penicillium oxalicum*			[205]					
*Penicillium oxalicum*	[83]		[83]	[83]				
*Penicillium rubens*	[209]							
*Penicillium sacculum*	[54,75]						[102]	
*Penicillium senticosum*	[83]		[83]	[83]				
*Penicillium* sp.	[102,206]						[102]	
*Periconia byssoides*	[83]			[83]				
*Pseudogymnoascus pannorum*	[54,75]						[102]	
*Schizophyllum commune*			[205]					
*Talaromyces fusiformis*	[83]		[83]	[83]				
*Talaromyces rugulosus*	[80]					[84]		
*Trichoderma lixii*				[83]				
*Trichoderma longibrachiatum*			[205]					
*Trichoderma viride*	[75,203]						[102]	
*Trichoderma reesei*			[205]					
*Trichoderma viridescens*	[210]		[210]					
*Trichoderma atroviride*			[205]					
*Trichoderma koningii*			[205]					
*Zalaria obscura*	[211]	[211]						
*Verrucocladosporium dirinae*	[207]							
*Vishniacozyma victoriae*	[212]							
*Wallemia* aff. *muriae*	[211]							

## Data Availability

Data are contained within the article.

## Appendix A

**Table A1:** Medium recipes to assess ligninocellulolytic activity on plate assays.

Enzymatic Activity	Medium Recipe g/L	Flooding Dye	References
Cellulase	KH_2_PO_4_ 0.5 g MgSO_4_ 0.25 g Cellulose 2 g Congo Red 0.2 g Gelatin 2 g Agar 15 g pH 6.8–7.2	-	[84,195,206]
Cellulase	NaNO_3_ 2 g KH_2_PO_4_ 1 g MgSO_4_·7H_2_O 0.5 g KCl 0.5 g FeSO_4_ 0.01 g agar, 20 g crystalline cellulose 10 g pH 5.5	-	[203]
Cellulase	Yeast Nitrogen Base 6.7 g glucose 1 g cellulose 5 g	0.3 g/L Congo Red solution	[192]
Endoglucanase	Peptone Yeast extract agar supplemented of azurine cross-linked hydroxyethyl-cellulose (AZCL-HE-Cellulose) 0.5 g		[197]
Endoglucanase	NaNO_3_ 2 g KH_2_PO_4_ 1 g MgSO_4_·7H_2_O 0.5 g KCl 0.5 g FeSO_4_ 0.01 g agar 20 g hydroxyethylcellulose dyed with Ostazin Brilliant Red H-3B (OBR) 2 g	-	[75,102]
Endoglucanase	K_2_HPO_4_ 0.5 g MgSO_4_ 0.25 g CMC sodium salt 1.88 g Congo red 0.2 g gelatin 2 g agar 17 g	-	[200]
Endoglucanase	(NH_4_)H_2_PO_4_ 1 g KCl 0.2 g MgSO_4_ 7H_2_O 1 g yeast extract 1 g CMC low-density 26 g bacteriological agar 3 g	Lugols’ iodine solution (15 min) and NaCl 0.9% destaining	[194,207]
Endoglucanase	Modified Czapek medium: NaNO_3_ 2 g KH_2_PO_4_ 1 g MgSO_4_·7H_2_O 0.5 g KCl 0.5 g FeSO_4_ 0.01 g CMC 10 g agar 20 g	0.1% Congo Red aqueous solution	[204]
Endoglucanase	NaNO_3_ 2 g K_2_HPO_4_ 1 g MgSO_4_ 0.5 g KCl 0.5 g peptone 0.2 g CMC sodium salt 2 g agar 17 g	Gram iodine (5 min)	[200]
Endoglucanase	NaNO_3_ 6.5 g K_2_HPO_4_ 6.5 g yeast extract 0.3 g KCl 6.5 g MgSO_4_ 3 g glucose 0.6 g CMC 10 g agar 17 g	1% Congo Red solution (30–60 min), destain NaCl 1 M (20 min)	[128]
Endoglucanase	YM (1:10) modified: Yeast extract 0.3 g Malt extract 0.3 g Peptone soybeans 0.5 g CMC 5 g Agar 15 g	0.1% Congo Red solution (15 min), destain NaCl 1 M	[212]
Endoglucanase	Modified Cz agar by adding CMC 4% carboxymethyl cellulose	0.3% Congo red solution (30 min), destain NaCl 1 M	[198,211]
Endoglucanase	Urea 0.3 g (NH_4_)_2_SO_4_ 1.4 g KH_2_PO_4_ 2 g CaCl_2_ 0.3 g MgSO_4_ 0.3 g yeast extract 0.25 g peptone 0.75 g FeSO_4_ 0.005 g CoCl_2_ 0.002 g MnSO_4_·H_2_O 0.0016 g ZnSO_4_ 0.0014 g agar 20 g CMC sodium salt 10 g pH adjusted to 5	0.1% Congo red solution (15 min) NaCl 1 M (15 min) destain	[193]
b-glucosidase	Cz agar with aesculin 3 g	-	[211]
b-glucosidase	YNB 6.7 g agar 20 g; arbutin (hydroquinone b-D-glucopyranoside) 4 g pH 5.0		[197]
Laccase	Cz Dox Agar with guaiacol 0.05% (w/v)		[199]
Laccase	PDA with guaiacol (0.02% v/v)		[201]
Laccase	NA with 0.01% guaiacol		[198]
Laccase	PDA with guaiacol 0.04% (v/v)		[200]
Laccase	Glucose 2 g peptone 1 g yeast extract 0.1 g agar 16 g Remazol Brilliant Blue R 0.4 g		[75,102]
Lignin peroxidase	Cz agar with azure B 0.0025% (*w*/*v*)		[199]
Lignin peroxidase	Glucose 2 g peptone 1 g yeast extract 0.1 g agar 16 g Azure B 0.2 g		[75,102]
Ligninase	(NH_4_)_2_SO_4_ 2 g K_2_HPO_4_ 0.5 g MgSO_4_ 7H_2_O 0.2 g agar 15 g Lignin 1 g		[83]
Mn peroxidase	Cz agar with 0.0025% Phenol Red (w/v)		[199]
Mn peroxidase	Glucose 2 g peptone 1 g yeast extract 0.1 g agar 16 g Phenol Red 0.1 g		[75,102]
Phenol oxidase activity	Cz agar with gallic acid 2 g.		[204]
Polyphenol oxidase activity	MEA with 1 mL tannic acid 1% (w/v)		[97]
Xylanase	PDA with xylan from beechwood 5 g		[83]
Xylanase	Cz agar with xylan 4% (w/w)		[198]

PDA: Potato dextrose agar; Cz: Czapek—Dox agar medium; NA: Nutrient Agar; MEA: Malt Extract Agar; YNB: Yeast nitrogen base.

**Table A2:** Basidiomycetous wood rotting fungi grouped by brown (B) and white rot (W) decaying trait according to previous studies [67,138,139,148].

Species	Rot	Species	Rot
*Amylocorticiellum molle* (Fr.) Spirin & Zmitr.	B	*Achanthophysellum fennicum* (Laurilia) Bernicchia & Gorjón	W
*Antrodia gossypium* (Speg.) Ryvarden	B	*Agrocybe cylindracea* (DC.) Maire	W
*Antrodia sinuosa* (Fr.) P. Karst.	B	*Alutaceodontia alutacea* (Fr.) Hjortstam & Ryvarden	W
*Antrodia* spp.	B	*Asterostroma cervicolor* (Berk. & Curtis) Massee	W
*Antrodia xantha* (Fr.) Ryvarden	B	*Asterostroma laxum* Bres.	W
*Brunneoporus malicola* (Berk. & M.A. Curtis) Audet	B	*Athelia decipiens* (Höhn. & Litsch.) J. Erikss.	W
*Cerinomyces pallidus* G.W. Martin	B	*Athelia epiphylia* (Höhn. & Litsch.) J. Erikss.	W
*Coniophora marmorata* Desm.	B	*Athelia neuhofii* (Bres.) Donk	W
*Coniophora arida* (Fr.) Karst	B	*Athelia pyriformis* (M. P. Christ) Jülich	W
*Coniophora olivacea* (Fr.) Karst	B	*Athelia* sp.	W
*Coniophora puteana* (Schumach.) P. Karst.	B	*Auricularia auricola-judae* (Bull.) J. Schröt.	W
*Crustoderma drynum* (Berk. & M. A. Curtis) Parmasto	B	*Auricularia mesenterica* (Dicks.) Pers.	W
*Cyanosporus caesius* (Schrad.) McGinty	B	*Bjerkandera adusta* (Willd.) P. Karst.	W
*Dacrymyces stillatus* Nees.	B	*Botryobasidium candicans* J. Erikss.	W
*Dacryobolus sudans* (Alb. & Shwein.) Fr.	B	*Botryobasidium laeve* (J. Erikss.) Parmasto	W
*Daedalea quercina* (L.) Pers.	B	*Botryobasidium obtusisporum* Johan Erikson	W
*Fibroporia vaillantii* (DC.) Parmastro	B	*Botryobasidium* spp.	W
*Fomitopsis pinicola* (Swartz) P. Karsten	B	*Botryobasidium subcoronatum* (Höhn. Litsch.) Donk	W
*Fomitopsis rosea* (Alb. & Schwein.) P. Karst.	B	*Botryobasidium vagum* (Berk. & M. A. Curtis) D. P. Rogers	W
*Gloeocystidiellum porosum* (Berk. & M. A. Curtis) Donk.	B	*Byssomerulius corium* (Pers.) Parmasto	W
*Gloeophyllum* spp.	B	*Ceraceomyces sublaevis* Bres.) Jülich	W
*Gloeophyllum abietinum* (Bull.) P. Karst.	B	*Ceriporia reticulata (Hoffm.) Domański*	W
*Gloeophyllum sepiarium* (Wulfen) P. Karst.	B	*Ceriporiopsis anereina* (Sommerf. Ff.) Dom	W
*Gloeophyllum trabeum* (Pers. Fr.) Murrill	B	*Ceriporiopsis excelsa* (S. Lundell) Parmasto	W
*Hydnomerulius pinastri* (Fr.) Jarosch & Besl	B	*Ceriporiopsis resinascens* (Romell) Domanski	W
*Laetiporus sulphureus* (Bull.) Murrill	B	*Ceriporiopsis* sp.	W
*Leucogyrophana mollusca* (Fr.) Pouzar	B	*Chondrostereum purpureum* (Pers.) Pouzar	W
*Leucogyrophana pseudomollusca* (Parmasto) Parm.	B	*Cinereomyces lindbladii* (Berk.) Jülich	W
*Meruliporia pulverulenta* (Sowerby) Zmitr., Kalinovskaya & Myasnikov	B	*Coprinellus micaceus* (Bull.) Vilgalys, Hopple & Jacq. Johnson	W
*Neoantrodia serialis* (Fr.) Audet	B	*Coprinus domesticus* (Bolton) Gray	W
*Neoantrodia serialis* (Fr.) Audet	B	*Coriolopsis galica* (Fr.) Ryvarden	W
*Neolentinus lepideus* (Fr.) Redhead & Ginns	B	*Corticium roseum* Pers.	W
*Odontia fibrosa* (Berk. & M.A. Curtis) Kõljalg,	B	*Crepidotus* spp.	W
*Oligoporus rennyi* (Berk. & Broome) Donk	B	*Crepidotus cesatii* (Rabenh.) Sacc.	W
*Phaeolus schweinitzii* (Fr.) Pat.	B	*Crepidotus mollis* (Schaeff.) Staude	W
*Postia caesia* (Schrad.) P. Karst.	B	*Cylindrobasidium evolvens* S. (Fr.) Fr.	W
*Postia fragilis* (Fr.) Jülich	B	*Cylindrobasidium laeve* (Pers.) Chamuris	W
*Postia guttulata* (Peck) Jülich	B	*Donkioporia expansa* (Desm.) Kotl. & Pouzar	W
*Postia stiptica* (Pers.) Julich	B	*Efibula tuberculata (P. Karst.) Zmitr. & Spirin*	W
*Postia subcaesia* (David) Jül	B	*Exidia glandulosa* (Bull.) Fr.	W
*Pycnoporellus fulgens* (Fr.) Donk	B	*Exidiopsis calcea* (Pers.) K. Wells	W
*Resinoporia sordida* (Ryvarden & Gilb.) Audet	B	*Exidiopsis* sp.	W
*Rhodonia placenta* (Fr.) Niemelä, K.H. Larss. & Schigel	B	*Fomes fomentarius* (L.) Fr.	W
*Serpula himantioides* (Fr.) P. Karst.	B	*Funalia gallica* Fr. Bondartsev & Singer	W
*Serpula lacrymans* (Wulfen) P. Karst.	B	*Fuscoporia contigua* (Pers.) G. Cunn.	W
*Tapinella panuoides* (Batsch) E. J. Gilbert	B	*Galerina hypnorum* (Shrank) Kühner	W
*Tomentella ferruginella Pers. Ex Pat.*	B	*Galerina* sp.	W
*Topinella panuoides* (Fr.) E.-J. Gilbert	B	*Ganoderma adspersum* (Schulzer) Donk	W
*Trechispora mollusca* (Pers.) Liberta	B	*Gloeocystidiellum convolvens* P. Karst. Donk	W
*Gloeocystidiellum luridum* (Bres.) Boidin	W	*Phlebia segregata* (Bourdt & Galzin) Parmasto	W
*Gloiothele citrina* (Pers.) Ginns & G. W. Freeman	W	*Phlebia tremellosa* (Schrad.) Nakasone & Burds.	W
*Grifola frondosa* (Dicks.) Gray	W	*Phlebiopsis gigantea* (Fr.) Jülich	W
*Hapalopilus nidulans* (Fr.) P. Karst.	W	*Phlebiopsis roumengueri* (Bresad.) Jülich & Stalp	W
*Haplotrichum capitatum* (Link) Link	W	*Physisporinus vitreus* (Pers.) P. Karst.	W
*Heterobasidion annosum* (Fr.) Bref.	W	*Pleurotus cornucopiae* (Paulet) Rolland	W
*Hymenochaetaceae*	W	*Pleurotus dryinus* (Pers.) P. Kumm	W
*Hymenochaete fuliginosa* (Pers.) Lév.	W	*Pleurotus ostreatus* (Jacq.) P. Kumm.	W
*Hyphoderma argillaceum* (Bres.) Donk	W	*Pleurotus pulmonarius* (Fr.) Quél.	W
*Hyphoderma obtusiforme* J. Erikss & Å. Strid	W	*Pluteus cervinus* (Schaeff.) P. Kumm.	W
*Hyphoderma obtusum* J. Erikss.	W	*Pluteus phlebophorus* (Ditmar)	W
*Hyphoderma occidentale* (D.P. Rogers) Boidin & Gilles	W	*Pluteus semibulbosus* (Lasch) Quél.	W
*Hyphoderma praetermissum* (P. Karst.) J. Erikss & Å. Strid	W	*Porodaedalea pini* (Brot.) Murrill	W
*Hyphoderma puberum* (Fr.) Walir.	W	*Radulomyces confluens* (Fr.) M.P. Christ.	W
*Hyphoderma setigerum* (Fr.) Donk	W	*Radulomyces confluens* (Fr.) M. P. Christ.	W
*Hyphodontia* sp.	W	*Resinicium bicolor* (Alb. & Schwein.) Parmasto	W
*Hyphodontia alutaria* (Burt) *I.* Erikss.	W	*Resinicium bicolor* (Alb. & Schwein.) Parmasto	W
*Hyphodontia arguta* (Fr.) J. Erikss.	W	*Resupinatus applicatus* (Batsch: Fr.) Gray	W
*Hyphodontia microspora* J. Erikss. & Hyortst	W	*Rhizochaete filamentosa* (Berk. & M.A. Curtis) Gresl., Nakasone & Rajchenb	W
*Hyphodontia pallidula* (Bres.) J. Erikss.	W	*Rhizochaete radicata* (Henn.) Gresl., Nakasone & Rajchenb	W
*Hypholoma fasciculare* (Huds.) P. Kumm.	W	*Schizophyllum commune* Fr.	W
*Hypochnicium bombycinum* (Sommerf.) J. Erikss.	W	*Schizopora paradoxa* (Schrad.) Donk	W
*Hypochnicium punctulatum* (Cooke) J. Erikss.	W	*Scytinostroma odoratum* (Fr.) Donk	W
*Irpex lacteus* (Fr.) Fr.	W	*Sistotrema brinkmannii* (Bres.) J. Erikss.	W
*Junghuhnia nitida* (Pers.) Ryvarden	W	*Sistotrema efibulatum* J. Erikss.	W
*Kneiffia subalutacea* (P. Karst.) Bres.	W	*Skeletocutis carneogrisea* A. David	W
*Kneiffiella floccosa* (Bourdot & Galzin) Jülich & Stalpers	W	*Skeletocutis percandida* (Malencon & Bertault) J. Keller	W
*Lopharia spedicea* (Pers.) Boidin	W	*Steccherinum bourdotii* Saliba & A. David	W
*Lyomyces crustosus* (Pers.) P. Karst.	W	*Stereum hirsutum* (Willd.) Pers.	W
*Lyomyces sambuci* (Pers.) P. Karst.	W	*Stereum rugosum* Pers.	W
*Marasmius torquescens* Quél.	W	*Stereum sanguinolentum* (Alb. & Schwein.) Fr.	W
*Mycena galericulata* (Scop.) Gray	W	*Trametes ochracea* (Pers.) Gilb. & Ryvarden	W
*Mycena silvae-nigrae* Maas Geest.	W	*Trametes hirsuta* (Wulfen) Lloyd	W
*Mycena* sp.	W	*Trametes trogii* (Berk.)	W
*Mycena stipata Maas* Geest. & Schwöbel	W	*Trametes versicolor* (L.) Lloyd	W
*Peniophora cinerea* (Pers.) Cooke	W	*Trechispora farinacea* (Pers.: Fr.) Liberta	W
*Peniophora incarnata* (Pers.) P. Karst.	W	*Trechispora invisitata* (H.J. Jacks.) Liberta	W
*Peniophora pithya* (Pers.) J. Erikss.	W	*Trechispora* sp.	W
*Peniophorella pubera* (Fr.) P. Karst.	W	*Tremella mesenterica* (Schaeff.) Retz.	W
*Peniophorella pubera* (Fr.) P. Karst	W	*Trichaptum abietinum* (Pers. ex J.F. Gmel.) Ryvarden	W
*Perenniporia medulla-panis* (Jacq.) Donk	W	*Trichaptum fusco-violaceum* (Ehrenb.) Ryvarden	W
*Phanerochaete calotricha* (Karst.) Erikss & Ryvarden	W	*Tubulicrinis calothrix* (Pat.) Donk	W
*Phanerochaete laevis* (Fr.) J. Erikss & Ryvarden	W	*Tubulicrinis glebulosum* (Fr.) Donk	W
*Phanerochaete sordida* (Karst.) Erikss & Ryvarden	W	*Tubulicrinis medius* (Bourdot & Galzin) Oberw.	W
*Phanerochaete* spp.	W	*Volvariella bombycina* (Schaeff.) Singer	W
*Phanerochaete velutina* (DC.) P. Karst.	W	*Xylodon asper* (Fr.) Hjortstam & Ryvarden	W
*Phellinus chrysoloma* (Fr.) Donk	W	*Xylodon brevisetus* (P. Karst.) Hjortstam & Ryvarden	W
*Phellinus punctatus* (P. Kurst.) Pilát	W	*Xylodon detriticus* (Bourdot) K.H. Larss., Viner & Spirin, in Viner, Spirin, Zíbarová & Larsson	W
*Phellopilus nigrolimitatus* (Romell) Niemelä, T. Wagner & M. Fisch.	W	*Xylodon nesporii* (Bres.) Hjortstam & Ryvarden	W
*Phlebia livida* (Pers.) Bres.	W		
